# Constraints on parton distribution functions and extraction of the strong coupling constant from the inclusive jet cross section in pp collisions at $$\sqrt{s} = 7$$$$\,\text {TeV}$$

**DOI:** 10.1140/epjc/s10052-015-3499-1

**Published:** 2015-06-26

**Authors:** V. Khachatryan, A. M. Sirunyan, A. Tumasyan, W. Adam, T. Bergauer, M. Dragicevic, J. Erö, M. Friedl, R. Frühwirth, V. M. Ghete, C. Hartl, N. Hörmann, J. Hrubec, M. Jeitler, W. Kiesenhofer, V. Knünz, M. Krammer, I. Krätschmer, D. Liko, I. Mikulec, D. Rabady, B. Rahbaran, H. Rohringer, R. Schöfbeck, J. Strauss, W. Treberer-Treberspurg, W. Waltenberger, C.-E. Wulz, V. Mossolov, N. Shumeiko, J. Suarez Gonzalez, S. Alderweireldt, M. Bansal, S. Bansal, T. Cornelis, E. A. De Wolf, X. Janssen, A. Knutsson, S. Luyckx, S. Ochesanu, R. Rougny, M. Van De Klundert, H. Van Haevermaet, P. Van Mechelen, N. Van Remortel, A. Van Spilbeeck, F. Blekman, S. Blyweert, J. D’Hondt, N. Daci, N. Heracleous, J. Keaveney, S. Lowette, M. Maes, A. Olbrechts, Q. Python, D. Strom, S. Tavernier, W. Van Doninck, P. Van Mulders, G. P. Van Onsem, I. Villella, C. Caillol, B. Clerbaux, G. De Lentdecker, D. Dobur, L. Favart, A. P. R. Gay, A. Grebenyuk, A. Léonard, A. Mohammadi, L. Perniè, T. Reis, T. Seva, L. Thomas, C. Vander Velde, P. Vanlaer, J. Wang, F. Zenoni, V. Adler, K. Beernaert, L. Benucci, A. Cimmino, S. Costantini, S. Crucy, S. Dildick, A. Fagot, G. Garcia, J. Mccartin, A. A. Ocampo Rios, D. Ryckbosch, S. Salva Diblen, M. Sigamani, N. Strobbe, F. Thyssen, M. Tytgat, E. Yazgan, N. Zaganidis, S. Basegmez, C. Beluffi, G. Bruno, R. Castello, A. Caudron, L. Ceard, G. G. Da Silveira, C. Delaere, T. du Pree, D. Favart, L. Forthomme, A. Giammanco, J. Hollar, A. Jafari, P. Jez, M. Komm, V. Lemaitre, C. Nuttens, D. Pagano, L. Perrini, A. Pin, K. Piotrzkowski, A. Popov, L. Quertenmont, M. Selvaggi, M. Vidal Marono, J. M. Vizan Garcia, N. Beliy, T. Caebergs, E. Daubie, G. H. Hammad, W. L. Aldá Júnior, G. A. Alves, L. Brito, M. Correa Martins Junior, T. Dos Reis Martins, C. Mora Herrera, M. E. Pol, W. Carvalho, J. Chinellato, A. Custódio, E. M. Da Costa, D. De Jesus Damiao, C. De Oliveira Martins, S. Fonseca De Souza, H. Malbouisson, D. Matos Figueiredo, L. Mundim, H. Nogima, W. L. Prado Da Silva, J. Santaolalla, A. Santoro, A. Sznajder, E. J. Tonelli Manganote, A. Vilela Pereira, C. A. Bernardes, S. Dogra, T. R. Fernandez Perez Tomei, E. M. Gregores, P. G. Mercadante, S. F. Novaes, Sandra S. Padula, A. Aleksandrov, V. Genchev, P. Iaydjiev, A. Marinov, S. Piperov, M. Rodozov, S. Stoykova, G. Sultanov, M. Vutova, A. Dimitrov, I. Glushkov, R. Hadjiiska, V. Kozhuharov, L. Litov, B. Pavlov, P. Petkov, J. G. Bian, G. M. Chen, H. S. Chen, M. Chen, R. Du, C. H. Jiang, R. Plestina, F. Romeo, J. Tao, Z. Wang, C. Asawatangtrakuldee, Y. Ban, Q. Li, S. Liu, Y. Mao, S. J. Qian, D. Wang, W. Zou, C. Avila, L. F. Chaparro Sierra, C. Florez, J. P. Gomez, B. Gomez Moreno, J. C. Sanabria, N. Godinovic, D. Lelas, D. Polic, I. Puljak, Z. Antunovic, M. Kovac, V. Brigljevic, K. Kadija, J. Luetic, D. Mekterovic, L. Sudic, A. Attikis, G. Mavromanolakis, J. Mousa, C. Nicolaou, F. Ptochos, P. A. Razis, M. Bodlak, M. Finger, M. Finger, Y. Assran, A. Ellithi Kamel, M. A. Mahmoud, A. Radi, M. Kadastik, M. Murumaa, M. Raidal, A. Tiko, P. Eerola, G. Fedi, M. Voutilainen, J. Härkönen, V. Karimäki, R. Kinnunen, M. J. Kortelainen, T. Lampén, K. Lassila-Perini, S. Lehti, T. Lindén, P. Luukka, T. Mäenpää, T. Peltola, E. Tuominen, J. Tuominiemi, E. Tuovinen, L. Wendland, J. Talvitie, T. Tuuva, M. Besancon, F. Couderc, M. Dejardin, D. Denegri, B. Fabbro, J. L. Faure, C. Favaro, F. Ferri, S. Ganjour, A. Givernaud, P. Gras, G. Hamel de Monchenault, P. Jarry, E. Locci, J. Malcles, J. Rander, A. Rosowsky, M. Titov, S. Baffioni, F. Beaudette, P. Busson, C. Charlot, T. Dahms, M. Dalchenko, L. Dobrzynski, N. Filipovic, A. Florent, R. Granier de Cassagnac, L. Mastrolorenzo, P. Miné, C. Mironov, I. N. Naranjo, M. Nguyen, C. Ochando, P. Paganini, S. Regnard, R. Salerno, J. B. Sauvan, Y. Sirois, C. Veelken, Y. Yilmaz, A. Zabi, J.-L. Agram, J. Andrea, A. Aubin, D. Bloch, J.-M. Brom, E. C. Chabert, C. Collard, E. Conte, J.-C. Fontaine, D. Gelé, U. Goerlach, C. Goetzmann, A.-C. Le Bihan, P. Van Hove, S. Gadrat, S. Beauceron, N. Beaupere, G. Boudoul, E. Bouvier, S. Brochet, C. A. Carrillo Montoya, J. Chasserat, R. Chierici, D. Contardo, P. Depasse, H. El Mamouni, J. Fan, J. Fay, S. Gascon, M. Gouzevitch, B. Ille, T. Kurca, M. Lethuillier, L. Mirabito, S. Perries, J. D. Ruiz Alvarez, D. Sabes, L. Sgandurra, V. Sordini, M. Vander Donckt, P. Verdier, S. Viret, H. Xiao, Z. Tsamalaidze, C. Autermann, S. Beranek, M. Bontenackels, M. Edelhoff, L. Feld, O. Hindrichs, K. Klein, A. Ostapchuk, A. Perieanu, F. Raupach, J. Sammet, S. Schael, H. Weber, B. Wittmer, V. Zhukov, M. Ata, M. Brodski, E. Dietz-Laursonn, D. Duchardt, M. Erdmann, R. Fischer, A. Güth, T. Hebbeker, C. Heidemann, K. Hoepfner, D. Klingebiel, S. Knutzen, P. Kreuzer, M. Merschmeyer, A. Meyer, P. Millet, M. Olschewski, K. Padeken, P. Papacz, H. Reithler, S. A. Schmitz, L. Sonnenschein, D. Teyssier, S. Thüer, M. Weber, V. Cherepanov, Y. Erdogan, G. Flügge, H. Geenen, M. Geisler, W. Haj Ahmad, A. Heister, F. Hoehle, B. Kargoll, T. Kress, Y. Kuessel, A. Künsken, J. Lingemann, A. Nowack, I. M. Nugent, L. Perchalla, O. Pooth, A. Stahl, I. Asin, N. Bartosik, J. Behr, W. Behrenhoff, U. Behrens, A. J. Bell, M. Bergholz, A. Bethani, K. Borras, A. Burgmeier, A. Cakir, L. Calligaris, A. Campbell, S. Choudhury, F. Costanza, C. Diez Pardos, S. Dooling, T. Dorland, G. Eckerlin, D. Eckstein, T. Eichhorn, G. Flucke, J. Garay Garcia, A. Geiser, P. Gunnellini, J. Hauk, M. Hempel, D. Horton, H. Jung, A. Kalogeropoulos, M. Kasemann, P. Katsas, J. Kieseler, C. Kleinwort, D. Krücker, W. Lange, J. Leonard, K. Lipka, A. Lobanov, W. Lohmann, B. Lutz, R. Mankel, I. Marfin, I.-A. Melzer-Pellmann, A. B. Meyer, G. Mittag, J. Mnich, A. Mussgiller, S. Naumann-Emme, A. Nayak, O. Novgorodova, E. Ntomari, H. Perrey, D. Pitzl, R. Placakyte, A. Raspereza, P. M. Ribeiro Cipriano, B. Roland, E. Ron, M. Ö. Sahin, J. Salfeld-Nebgen, P. Saxena, R. Schmidt, T. Schoerner-Sadenius, M. Schröder, C. Seitz, S. Spannagel, A. D. R. Vargas Trevino, R. Walsh, C. Wissing, M. Aldaya Martin, V. Blobel, M. Centis Vignali, A. R. Draeger, J. Erfle, E. Garutti, K. Goebel, M. Görner, J. Haller, M. Hoffmann, R. S. Höing, H. Kirschenmann, R. Klanner, R. Kogler, J. Lange, T. Lapsien, T. Lenz, I. Marchesini, J. Ott, T. Peiffer, N. Pietsch, J. Poehlsen, T. Poehlsen, D. Rathjens, C. Sander, H. Schettler, P. Schleper, E. Schlieckau, A. Schmidt, M. Seidel, V. Sola, H. Stadie, G. Steinbrück, D. Troendle, E. Usai, L. Vanelderen, A. Vanhoefer, C. Barth, C. Baus, J. Berger, C. Böser, E. Butz, T. Chwalek, W. De Boer, A. Descroix, A. Dierlamm, M. Feindt, F. Frensch, M. Giffels, F. Hartmann, T. Hauth, U. Husemann, I. Katkov, A. Kornmayer, E. Kuznetsova, P. Lobelle Pardo, M. U. Mozer, Th. Müller, A. Nürnberg, G. Quast, K. Rabbertz, F. Ratnikov, S. Röcker, G. Sieber, H. J. Simonis, F. M. Stober, R. Ulrich, J. Wagner-Kuhr, S. Wayand, T. Weiler, R. Wolf, G. Anagnostou, G. Daskalakis, T. Geralis, V. A. Giakoumopoulou, A. Kyriakis, D. Loukas, A. Markou, C. Markou, A. Psallidas, I. Topsis-Giotis, A. Agapitos, S. Kesisoglou, A. Panagiotou, N. Saoulidou, E. Stiliaris, X. Aslanoglou, I. Evangelou, G. Flouris, C. Foudas, P. Kokkas, N. Manthos, I. Papadopoulos, E. Paradas, G. Bencze, C. Hajdu, P. Hidas, D. Horvath, F. Sikler, V. Veszpremi, G. Vesztergombi, A. J. Zsigmond, N. Beni, S. Czellar, J. Karancsi, J. Molnar, J. Palinkas, Z. Szillasi, A. Makovec, P. Raics, Z. L. Trocsanyi, B. Ujvari, S. K. Swain, S. B. Beri, V. Bhatnagar, R. Gupta, U. Bhawandeep, A. K. Kalsi, M. Kaur, R. Kumar, M. Mittal, N. Nishu, J. B. Singh, Ashok Kumar, Arun Kumar, S. Ahuja, A. Bhardwaj, B. C. Choudhary, A. Kumar, S. Malhotra, M. Naimuddin, K. Ranjan, V. Sharma, S. Banerjee, S. Bhattacharya, K. Chatterjee, S. Dutta, B. Gomber, Sa. Jain, Sh. Jain, R. Khurana, A. Modak, S. Mukherjee, D. Roy, S. Sarkar, M. Sharan, A. Abdulsalam, D. Dutta, S. Kailas, V. Kumar, A. K. Mohanty, L. M. Pant, P. Shukla, A. Topkar, T. Aziz, S. Banerjee, S. Bhowmik, R. M. Chatterjee, R. K. Dewanjee, S. Dugad, S. Ganguly, S. Ghosh, M. Guchait, A. Gurtu, G. Kole, S. Kumar, M. Maity, G. Majumder, K. Mazumdar, G. B. Mohanty, B. Parida, K. Sudhakar, N. Wickramage, H. Bakhshiansohi, H. Behnamian, S. M. Etesami, A. Fahim, R. Goldouzian, M. Khakzad, M. Mohammadi Najafabadi, M. Naseri, S. Paktinat Mehdiabadi, F. Rezaei Hosseinabadi, B. Safarzadeh, M. Zeinali, M. Felcini, M. Grunewald, M. Abbrescia, C. Calabria, S. S. Chhibra, A. Colaleo, D. Creanza, N. De Filippis, M. De Palma, L. Fiore, G. Iaselli, G. Maggi, M. Maggi, S. My, S. Nuzzo, A. Pompili, G. Pugliese, R. Radogna, G. Selvaggi, A. Sharma, L. Silvestris, R. Venditti, G. Zito, G. Abbiendi, A. C. Benvenuti, D. Bonacorsi, S. Braibant-Giacomelli, L. Brigliadori, R. Campanini, P. Capiluppi, A. Castro, F. R. Cavallo, G. Codispoti, M. Cuffiani, G. M. Dallavalle, F. Fabbri, A. Fanfani, D. Fasanella, P. Giacomelli, C. Grandi, L. Guiducci, S. Marcellini, G. Masetti, A. Montanari, F. L. Navarria, A. Perrotta, A. M. Rossi, F. Primavera, T. Rovelli, G. P. Siroli, N. Tosi, R. Travaglini, S. Albergo, G. Cappello, M. Chiorboli, S. Costa, F. Giordano, R. Potenza, A. Tricomi, C. Tuve, G. Barbagli, V. Ciulli, C. Civinini, R. D’Alessandro, E. Focardi, E. Gallo, S. Gonzi, V. Gori, P. Lenzi, M. Meschini, S. Paoletti, G. Sguazzoni, A. Tropiano, L. Benussi, S. Bianco, F. Fabbri, D. Piccolo, R. Ferretti, F. Ferro, M. Lo Vetere, E. Robutti, S. Tosi, M. E. Dinardo, S. Fiorendi, S. Gennai, R. Gerosa, A. Ghezzi, P. Govoni, M. T. Lucchini, S. Malvezzi, R. A. Manzoni, A. Martelli, B. Marzocchi, D. Menasce, L. Moroni, M. Paganoni, D. Pedrini, S. Ragazzi, N. Redaelli, T. Tabarelli de Fatis, S. Buontempo, N. Cavallo, S. Di Guida, F. Fabozzi, A. O. M. Iorio, L. Lista, S. Meola, M. Merola, P. Paolucci, P. Azzi, N. Bacchetta, M. Biasotto, D. Bisello, A. Branca, R. Carlin, P. Checchia, M. Dall’Osso, T. Dorigo, U. Dosselli, M. Galanti, F. Gasparini, U. Gasparini, P. Giubilato, F. Gonella, A. Gozzelino, K. Kanishchev, S. Lacaprara, M. Margoni, F. Montecassiano, J. Pazzini, N. Pozzobon, P. Ronchese, M. Tosi, S. Vanini, S. Ventura, A. Zucchetta, M. Gabusi, S. P. Ratti, V. Re, C. Riccardi, P. Salvini, P. Vitulo, M. Biasini, G. M. Bilei, D. Ciangottini, L. Fanò, P. Lariccia, G. Mantovani, M. Menichelli, A. Saha, A. Santocchia, A. Spiezia, K. Androsov, P. Azzurri, G. Bagliesi, J. Bernardini, T. Boccali, G. Broccolo, R. Castaldi, M. A. Ciocci, R. Dell’Orso, S. Donato, G. Fedi, F. Fiori, L. Foà, A. Giassi, M. T. Grippo, F. Ligabue, T. Lomtadze, L. Martini, A. Messineo, C. S. Moon, F. Palla, A. Rizzi, A. Savoy-Navarro, A. T. Serban, P. Spagnolo, P. Squillacioti, R. Tenchini, G. Tonelli, A. Venturi, P. G. Verdini, C. Vernieri, L. Barone, F. Cavallari, G. D’imperio, D. Del Re, M. Diemoz, C. Jorda, E. Longo, F. Margaroli, P. Meridiani, F. Micheli, S. Nourbakhsh, G. Organtini, R. Paramatti, S. Rahatlou, C. Rovelli, F. Santanastasio, L. Soffi, P. Traczyk, N. Amapane, R. Arcidiacono, S. Argiro, M. Arneodo, R. Bellan, C. Biino, N. Cartiglia, S. Casasso, M. Costa, A. Degano, N. Demaria, L. Finco, C. Mariotti, S. Maselli, E. Migliore, V. Monaco, M. Musich, M. M. Obertino, G. Ortona, L. Pacher, N. Pastrone, M. Pelliccioni, G. L. Pinna Angioni, A. Potenza, A. Romero, M. Ruspa, R. Sacchi, A. Solano, A. Staiano, U. Tamponi, S. Belforte, V. Candelise, M. Casarsa, F. Cossutti, G. Della Ricca, B. Gobbo, C. La Licata, M. Marone, A. Schizzi, T. Umer, A. Zanetti, S. Chang, T. A. Kropivnitskaya, S. K. Nam, D. H. Kim, G. N. Kim, M. S. Kim, M. S. Kim, D. J. Kong, S. Lee, Y. D. Oh, H. Park, A. Sakharov, D. C. Son, T. J. Kim, J. Y. Kim, S. Song, S. Choi, D. Gyun, B. Hong, M. Jo, H. Kim, Y. Kim, B. Lee, K. S. Lee, S. K. Park, Y. Roh, M. Choi, J. H. Kim, I. C. Park, G. Ryu, M. S. Ryu, Y. Choi, Y. K. Choi, J. Goh, D. Kim, E. Kwon, J. Lee, H. Seo, I. Yu, A. Juodagalvis, J. R. Komaragiri, M. A. B. Md Ali, E. Casimiro Linares, H. Castilla-Valdez, E. De La Cruz-Burelo, I. Heredia-de La Cruz, A. Hernandez-Almada, R. Lopez-Fernandez, A. Sanchez-Hernandez, S. Carrillo Moreno, F. Vazquez Valencia, I. Pedraza, H. A. Salazar Ibarguen, A. Morelos Pineda, D. Krofcheck, P. H. Butler, S. Reucroft, A. Ahmad, M. Ahmad, Q. Hassan, H. R. Hoorani, W. A. Khan, T. Khurshid, M. Shoaib, H. Bialkowska, M. Bluj, B. Boimska, T. Frueboes, M. Górski, M. Kazana, K. Nawrocki, K. Romanowska-Rybinska, M. Szleper, P. Zalewski, G. Brona, K. Bunkowski, M. Cwiok, W. Dominik, K. Doroba, A. Kalinowski, M. Konecki, J. Krolikowski, M. Misiura, M. Olszewski, W. Wolszczak, P. Bargassa, C. Beir ao Da Cruz E Silva, P. Faccioli, P. G. Ferreira Parracho, M. Gallinaro, L. Lloret Iglesias, F. Nguyen, J. Rodrigues Antunes, J. Seixas, J. Varela, P. Vischia, S. Afanasiev, P. Bunin, M. Gavrilenko, I. Golutvin, I. Gorbunov, A. Kamenev, V. Karjavin, V. Konoplyanikov, A. Lanev, A. Malakhov, V. Matveev, P. Moisenz, V. Palichik, V. Perelygin, S. Shmatov, N. Skatchkov, V. Smirnov, A. Zarubin, V. Golovtsov, Y. Ivanov, V. Kim, P. Levchenko, V. Murzin, V. Oreshkin, I. Smirnov, V. Sulimov, L. Uvarov, S. Vavilov, A. Vorobyev, An. Vorobyev, Yu. Andreev, A. Dermenev, S. Gninenko, N. Golubev, M. Kirsanov, N. Krasnikov, A. Pashenkov, D. Tlisov, A. Toropin, V. Epshteyn, V. Gavrilov, N. Lychkovskaya, V. Popov, I. Pozdnyakov, G. Safronov, S. Semenov, A. Spiridonov, V. Stolin, E. Vlasov, A. Zhokin, V. Andreev, M. Azarkin, I. Dremin, M. Kirakosyan, A. Leonidov, G. Mesyats, S. V. Rusakov, A. Vinogradov, A. Belyaev, E. Boos, M. Dubinin, L. Dudko, A. Ershov, A. Gribushin, V. Klyukhin, O. Kodolova, I. Lokhtin, S. Obraztsov, S. Petrushanko, V. Savrin, A. Snigirev, I. Azhgirey, I. Bayshev, S. Bitioukov, V. Kachanov, A. Kalinin, D. Konstantinov, V. Krychkine, V. Petrov, R. Ryutin, A. Sobol, L. Tourtchanovitch, S. Troshin, N. Tyurin, A. Uzunian, A. Volkov, P. Adzic, M. Ekmedzic, J. Milosevic, V. Rekovic, J. Alcaraz Maestre, C. Battilana, E. Calvo, M. Cerrada, M. Chamizo Llatas, N. Colino, B. De La Cruz, A. Delgado Peris, D. Domínguez Vázquez, A. Escalante Del Valle, C. Fernandez Bedoya, J. P. Fernández Ramos, J. Flix, M. C. Fouz, P. Garcia-Abia, O. Gonzalez Lopez, S. Goy Lopez, J. M. Hernandez, M. I. Josa, E. Navarro De Martino, A. Pérez-Calero Yzquierdo, J. Puerta Pelayo, A. Quintario Olmeda, I. Redondo, L. Romero, M. S. Soares, C. Albajar, J. F. de Trocóniz, M. Missiroli, D. Moran, H. Brun, J. Cuevas, J. Fernandez Menendez, S. Folgueras, I. Gonzalez Caballero, J. A. Brochero Cifuentes, I. J. Cabrillo, A. Calderon, J. Duarte Campderros, M. Fernandez, G. Gomez, A. Graziano, A. Lopez Virto, J. Marco, R. Marco, C. Martinez Rivero, F. Matorras, F. J. Munoz Sanchez, J. Piedra Gomez, T. Rodrigo, A. Y. Rodríguez-Marrero, A. Ruiz-Jimeno, L. Scodellaro, I. Vila, R. Vilar Cortabitarte, D. Abbaneo, E. Auffray, G. Auzinger, M. Bachtis, P. Baillon, A. H. Ball, D. Barney, A. Benaglia, J. Bendavid, L. Benhabib, J. F. Benitez, C. Bernet, P. Bloch, A. Bocci, A. Bonato, O. Bondu, C. Botta, H. Breuker, T. Camporesi, G. Cerminara, S. Colafranceschi, M. D’Alfonso, D. d’Enterria, A. Dabrowski, A. David, F. De Guio, A. De Roeck, S. De Visscher, E. Di Marco, M. Dobson, M. Dordevic, N. Dupont-Sagorin, A. Elliott-Peisert, J. Eugster, G. Franzoni, W. Funk, D. Gigi, K. Gill, D. Giordano, M. Girone, F. Glege, R. Guida, S. Gundacker, M. Guthoff, J. Hammer, M. Hansen, P. Harris, J. Hegeman, V. Innocente, P. Janot, K. Kousouris, K. Krajczar, P. Lecoq, C. Lourenço, N. Magini, L. Malgeri, M. Mannelli, J. Marrouche, L. Masetti, F. Meijers, S. Mersi, E. Meschi, F. Moortgat, S. Morovic, M. Mulders, P. Musella, L. Orsini, L. Pape, E. Perez, L. Perrozzi, A. Petrilli, G. Petrucciani, A. Pfeiffer, M. Pierini, M. Pimiä, D. Piparo, M. Plagge, A. Racz, G. Rolandi, M. Rovere, H. Sakulin, C. Schäfer, C. Schwick, A. Sharma, P. Siegrist, P. Silva, M. Simon, P. Sphicas, D. Spiga, J. Steggemann, B. Stieger, M. Stoye, Y. Takahashi, D. Treille, A. Tsirou, G. I. Veres, N. Wardle, H. K. Wöhri, H. Wollny, W. D. Zeuner, W. Bertl, K. Deiters, W. Erdmann, R. Horisberger, Q. Ingram, H. C. Kaestli, D. Kotlinski, U. Langenegger, D. Renker, T. Rohe, F. Bachmair, L. Bäni, L. Bianchini, M. A. Buchmann, B. Casal, N. Chanon, G. Dissertori, M. Dittmar, M. Donegà, M. Dünser, P. Eller, C. Grab, D. Hits, J. Hoss, W. Lustermann, B. Mangano, A. C. Marini, P. Martinez Ruiz del Arbol, M. Masciovecchio, D. Meister, N. Mohr, C. Nägeli, F. Nessi-Tedaldi, F. Pandolfi, F. Pauss, M. Peruzzi, M. Quittnat, L. Rebane, M. Rossini, A. Starodumov, M. Takahashi, K. Theofilatos, R. Wallny, H. A. Weber, C. Amsler, M. F. Canelli, V. Chiochia, A. De Cosa, A. Hinzmann, T. Hreus, B. Kilminster, C. Lange, B. Millan Mejias, J. Ngadiuba, P. Robmann, F. J. Ronga, S. Taroni, M. Verzetti, Y. Yang, M. Cardaci, K. H. Chen, C. Ferro, C. M. Kuo, W. Lin, Y. J. Lu, R. Volpe, S. S. Yu, P. Chang, Y. H. Chang, Y. W. Chang, Y. Chao, K. F. Chen, P. H. Chen, C. Dietz, U. Grundler, W.-S. Hou, K. Y. Kao, Y. J. Lei, Y. F. Liu, R.-S. Lu, D. Majumder, E. Petrakou, Y. M. Tzeng, R. Wilken, B. Asavapibhop, G. Singh, N. Srimanobhas, N. Suwonjandee, A. Adiguzel, M. N. Bakirci, S. Cerci, C. Dozen, I. Dumanoglu, E. Eskut, S. Girgis, G. Gokbulut, E. Gurpinar, I. Hos, E. E. Kangal, A. Kayis Topaksu, G. Onengut, K. Ozdemir, S. Ozturk, A. Polatoz, D. Sunar Cerci, B. Tali, H. Topakli, M. Vergili, I. V. Akin, B. Bilin, S. Bilmis, H. Gamsizkan, B. Isildak, G. Karapinar, K. Ocalan, S. Sekmen, U. E. Surat, M. Yalvac, M. Zeyrek, E. A. Albayrak, E. Gülmez, B. Isildak, M. Kaya, O. Kaya, T. Yetkin, K. Cankocak, F. I. Vardarlı, L. Levchuk, P. Sorokin, J. J. Brooke, E. Clement, D. Cussans, H. Flacher, J. Goldstein, M. Grimes, G. P. Heath, H. F. Heath, J. Jacob, L. Kreczko, C. Lucas, Z. Meng, D. M. Newbold, S. Paramesvaran, A. Poll, S. Senkin, V. J. Smith, T. Williams, K. W. Bell, A. Belyaev, C. Brew, R. M. Brown, D. J. A. Cockerill, J. A. Coughlan, K. Harder, S. Harper, E. Olaiya, D. Petyt, C. H. Shepherd-Themistocleous, A. Thea, I. R. Tomalin, W. J. Womersley, S. D. Worm, M. Baber, R. Bainbridge, O. Buchmuller, D. Burton, D. Colling, N. Cripps, M. Cutajar, P. Dauncey, G. Davies, M. Della Negra, P. Dunne, W. Ferguson, J. Fulcher, D. Futyan, A. Gilbert, G. Hall, G. Iles, M. Jarvis, G. Karapostoli, M. Kenzie, R. Lane, R. Lucas, L. Lyons, A.-M. Magnan, S. Malik, B. Mathias, J. Nash, A. Nikitenko, J. Pela, M. Pesaresi, K. Petridis, D. M. Raymond, S. Rogerson, A. Rose, C. Seez, P. Sharp, A. Tapper, M. Vazquez Acosta, T. Virdee, S. C. Zenz, J. E. Cole, P. R. Hobson, A. Khan, P. Kyberd, D. Leggat, D. Leslie, W. Martin, I. D. Reid, P. Symonds, L. Teodorescu, M. Turner, J. Dittmann, K. Hatakeyama, A. Kasmi, H. Liu, T. Scarborough, O. Charaf, S. I. Cooper, C. Henderson, P. Rumerio, A. Avetisyan, T. Bose, C. Fantasia, P. Lawson, C. Richardson, J. Rohlf, J. St. John, L. Sulak, J. Alimena, E. Berry, S. Bhattacharya, G. Christopher, D. Cutts, Z. Demiragli, N. Dhingra, A. Ferapontov, A. Garabedian, U. Heintz, G. Kukartsev, E. Laird, G. Landsberg, M. Luk, M. Narain, M. Segala, T. Sinthuprasith, T. Speer, J. Swanson, R. Breedon, G. Breto, M. Calderon De La Barca Sanchez, S. Chauhan, M. Chertok, J. Conway, R. Conway, P. T. Cox, R. Erbacher, M. Gardner, W. Ko, R. Lander, T. Miceli, M. Mulhearn, D. Pellett, J. Pilot, F. Ricci-Tam, M. Searle, S. Shalhout, J. Smith, M. Squires, D. Stolp, M. Tripathi, S. Wilbur, R. Yohay, R. Cousins, P. Everaerts, C. Farrell, J. Hauser, M. Ignatenko, G. Rakness, E. Takasugi, V. Valuev, M. Weber, K. Burt, R. Clare, J. Ellison, J. W. Gary, G. Hanson, J. Heilman, M. Ivova Rikova, P. Jandir, E. Kennedy, F. Lacroix, O. R. Long, A. Luthra, M. Malberti, M. Olmedo Negrete, A. Shrinivas, S. Sumowidagdo, S. Wimpenny, J. G. Branson, G. B. Cerati, S. Cittolin, R. T. D’Agnolo, A. Holzner, R. Kelley, D. Klein, J. Letts, I. Macneill, D. Olivito, S. Padhi, C. Palmer, M. Pieri, M. Sani, V. Sharma, S. Simon, E. Sudano, M. Tadel, Y. Tu, A. Vartak, C. Welke, F. Würthwein, A. Yagil, D. Barge, J. Bradmiller-Feld, C. Campagnari, T. Danielson, A. Dishaw, V. Dutta, K. Flowers, M. Franco Sevilla, P. Geffert, C. George, F. Golf, L. Gouskos, J. Incandela, C. Justus, N. Mccoll, J. Richman, D. Stuart, W. To, C. West, J. Yoo, A. Apresyan, A. Bornheim, J. Bunn, Y. Chen, J. Duarte, A. Mott, H. B. Newman, C. Pena, C. Rogan, M. Spiropulu, V. Timciuc, J. R. Vlimant, R. Wilkinson, S. Xie, R. Y. Zhu, V. Azzolini, A. Calamba, B. Carlson, T. Ferguson, Y. Iiyama, M. Paulini, J. Russ, H. Vogel, I. Vorobiev, J. P. Cumalat, W. T. Ford, A. Gaz, M. Krohn, E. Luiggi Lopez, U. Nauenberg, J. G. Smith, K. Stenson, K. A. Ulmer, S. R. Wagner, J. Alexander, A. Chatterjee, J. Chaves, J. Chu, S. Dittmer, N. Eggert, N. Mirman, G. Nicolas Kaufman, J. R. Patterson, A. Ryd, E. Salvati, L. Skinnari, W. Sun, W. D. Teo, J. Thom, J. Thompson, J. Tucker, Y. Weng, L. Winstrom, P. Wittich, D. Winn, S. Abdullin, M. Albrow, J. Anderson, G. Apollinari, L. A. T. Bauerdick, A. Beretvas, J. Berryhill, P. C. Bhat, G. Bolla, K. Burkett, J. N. Butler, H. W. K. Cheung, F. Chlebana, S. Cihangir, V. D. Elvira, I. Fisk, J. Freeman, Y. Gao, E. Gottschalk, L. Gray, D. Green, S. Grünendahl, O. Gutsche, J. Hanlon, D. Hare, R. M. Harris, J. Hirschauer, B. Hooberman, S. Jindariani, M. Johnson, U. Joshi, K. Kaadze, B. Klima, B. Kreis, S. Kwan, J. Linacre, D. Lincoln, R. Lipton, T. Liu, J. Lykken, K. Maeshima, J. M. Marraffino, V. I. Martinez Outschoorn, S. Maruyama, D. Mason, P. McBride, P. Merkel, K. Mishra, S. Mrenna, Y. Musienko, S. Nahn, C. Newman-Holmes, V. O’Dell, O. Prokofyev, E. Sexton-Kennedy, S. Sharma, A. Soha, W. J. Spalding, L. Spiegel, L. Taylor, S. Tkaczyk, N. V. Tran, L. Uplegger, E. W. Vaandering, R. Vidal, A. Whitbeck, J. Whitmore, F. Yang, D. Acosta, P. Avery, P. Bortignon, D. Bourilkov, M. Carver, T. Cheng, D. Curry, S. Das, M. De Gruttola, G. P. Di Giovanni, R. D. Field, M. Fisher, I. K. Furic, J. Hugon, J. Konigsberg, A. Korytov, T. Kypreos, J. F. Low, K. Matchev, P. Milenovic, G. Mitselmakher, L. Muniz, A. Rinkevicius, L. Shchutska, M. Snowball, D. Sperka, J. Yelton, M. Zakaria, S. Hewamanage, S. Linn, P. Markowitz, G. Martinez, J. L. Rodriguez, T. Adams, A. Askew, J. Bochenek, B. Diamond, J. Haas, S. Hagopian, V. Hagopian, K. F. Johnson, H. Prosper, V. Veeraraghavan, M. Weinberg, M. M. Baarmand, M. Hohlmann, H. Kalakhety, F. Yumiceva, M. R. Adams, L. Apanasevich, V. E. Bazterra, D. Berry, R. R. Betts, I. Bucinskaite, R. Cavanaugh, O. Evdokimov, L. Gauthier, C. E. Gerber, D. J. Hofman, S. Khalatyan, P. Kurt, D. H. Moon, C. O’Brien, C. Silkworth, P. Turner, N. Varelas, B. Bilki, W. Clarida, K. Dilsiz, F. Duru, M. Haytmyradov, J.-P. Merlo, H. Mermerkaya, A. Mestvirishvili, A. Moeller, J. Nachtman, H. Ogul, Y. Onel, F. Ozok, A. Penzo, R. Rahmat, S. Sen, P. Tan, E. Tiras, J. Wetzel, K. Yi, B. A. Barnett, B. Blumenfeld, S. Bolognesi, D. Fehling, A. V. Gritsan, P. Maksimovic, C. Martin, M. Swartz, P. Baringer, A. Bean, G. Benelli, C. Bruner, R. P. Kenny, M. Malek, M. Murray, D. Noonan, S. Sanders, J. Sekaric, R. Stringer, Q. Wang, J. S. Wood, I. Chakaberia, A. Ivanov, S. Khalil, M. Makouski, Y. Maravin, L. K. Saini, S. Shrestha, N. Skhirtladze, I. Svintradze, J. Gronberg, D. Lange, F. Rebassoo, D. Wright, A. Baden, A. Belloni, B. Calvert, S. C. Eno, J. A. Gomez, N. J. Hadley, R. G. Kellogg, T. Kolberg, Y. Lu, M. Marionneau, A. C. Mignerey, K. Pedro, A. Skuja, M. B. Tonjes, S. C. Tonwar, A. Apyan, R. Barbieri, G. Bauer, W. Busza, I. A. Cali, M. Chan, L. Di Matteo, G. Gomez Ceballos, M. Goncharov, D. Gulhan, M. Klute, Y. S. Lai, Y.-J. Lee, A. Levin, P. D. Luckey, T. Ma, C. Paus, D. Ralph, C. Roland, G. Roland, G. S. F. Stephans, F. Stöckli, K. Sumorok, D. Velicanu, J. Veverka, B. Wyslouch, M. Yang, M. Zanetti, V. Zhukova, B. Dahmes, A. Gude, S. C. Kao, K. Klapoetke, Y. Kubota, J. Mans, N. Pastika, R. Rusack, A. Singovsky, N. Tambe, J. Turkewitz, J. G. Acosta, S. Oliveros, E. Avdeeva, K. Bloom, S. Bose, D. R. Claes, A. Dominguez, R. Gonzalez Suarez, J. Keller, D. Knowlton, I. Kravchenko, J. Lazo-Flores, S. Malik, F. Meier, G. R. Snow, M. Zvada, J. Dolen, A. Godshalk, I. Iashvili, A. Kharchilava, A. Kumar, S. Rappoccio, G. Alverson, E. Barberis, D. Baumgartel, M. Chasco, J. Haley, A. Massironi, D. M. Morse, D. Nash, T. Orimoto, D. Trocino, R. J. Wang, D. Wood, J. Zhang, K. A. Hahn, A. Kubik, N. Mucia, N. Odell, B. Pollack, A. Pozdnyakov, M. Schmitt, S. Stoynev, K. Sung, M. Velasco, S. Won, A. Brinkerhoff, K. M. Chan, A. Drozdetskiy, M. Hildreth, C. Jessop, D. J. Karmgard, N. Kellams, K. Lannon, W. Luo, S. Lynch, N. Marinelli, T. Pearson, M. Planer, R. Ruchti, N. Valls, M. Wayne, M. Wolf, A. Woodard, L. Antonelli, J. Brinson, B. Bylsma, L. S. Durkin, S. Flowers, A. Hart, C. Hill, R. Hughes, K. Kotov, T. Y. Ling, D. Puigh, M. Rodenburg, G. Smith, B. L. Winer, H. Wolfe, H. W. Wulsin, O. Driga, P. Elmer, J. Hardenbrook, P. Hebda, A. Hunt, S. A. Koay, P. Lujan, D. Marlow, T. Medvedeva, M. Mooney, J. Olsen, P. Piroué, X. Quan, H. Saka, D. Stickland, C. Tully, J. S. Werner, A. Zuranski, E. Brownson, H. Mendez, J. E. Ramirez Vargas, V. E. Barnes, D. Benedetti, D. Bortoletto, M. De Mattia, L. Gutay, Z. Hu, M. K. Jha, M. Jones, K. Jung, M. Kress, N. Leonardo, D. Lopes Pegna, V. Maroussov, D. H. Miller, N. Neumeister, B. C. Radburn-Smith, X. Shi, I. Shipsey, D. Silvers, A. Svyatkovskiy, F. Wang, W. Xie, L. Xu, H. D. Yoo, J. Zablocki, Y. Zheng, N. Parashar, J. Stupak, A. Adair, B. Akgun, K. M. Ecklund, F. J. M. Geurts, W. Li, B. Michlin, B. P. Padley, R. Redjimi, J. Roberts, J. Zabel, B. Betchart, A. Bodek, R. Covarelli, P. de Barbaro, R. Demina, Y. Eshaq, T. Ferbel, A. Garcia-Bellido, P. Goldenzweig, J. Han, A. Harel, A. Khukhunaishvili, G. Petrillo, D. Vishnevskiy, R. Ciesielski, L. Demortier, K. Goulianos, G. Lungu, C. Mesropian, S. Arora, A. Barker, J. P. Chou, C. Contreras-Campana, E. Contreras-Campana, D. Duggan, D. Ferencek, Y. Gershtein, R. Gray, E. Halkiadakis, D. Hidas, S. Kaplan, A. Lath, S. Panwalkar, M. Park, R. Patel, S. Salur, S. Schnetzer, S. Somalwar, R. Stone, S. Thomas, P. Thomassen, M. Walker, K. Rose, S. Spanier, A. York, O. Bouhali, A. Castaneda Hernandez, R. Eusebi, W. Flanagan, J. Gilmore, T. Kamon, V. Khotilovich, V. Krutelyov, R. Montalvo, I. Osipenkov, Y. Pakhotin, A. Perloff, J. Roe, A. Rose, A. Safonov, T. Sakuma, I. Suarez, A. Tatarinov, N. Akchurin, C. Cowden, J. Damgov, C. Dragoiu, P. R. Dudero, J. Faulkner, K. Kovitanggoon, S. Kunori, S. W. Lee, T. Libeiro, I. Volobouev, E. Appelt, A. G. Delannoy, S. Greene, A. Gurrola, W. Johns, C. Maguire, Y. Mao, A. Melo, M. Sharma, P. Sheldon, B. Snook, S. Tuo, J. Velkovska, M. W. Arenton, S. Boutle, B. Cox, B. Francis, J. Goodell, R. Hirosky, A. Ledovskoy, H. Li, C. Lin, C. Neu, J. Wood, C. Clarke, R. Harr, P. E. Karchin, C. Kottachchi Kankanamge Don, P. Lamichhane, J. Sturdy, D. A. Belknap, D. Carlsmith, M. Cepeda, S. Dasu, L. Dodd, S. Duric, E. Friis, R. Hall-Wilton, M. Herndon, A. Hervé, P. Klabbers, A. Lanaro, C. Lazaridis, A. Levine, R. Loveless, A. Mohapatra, I. Ojalvo, T. Perry, G. A. Pierro, G. Polese, I. Ross, T. Sarangi, A. Savin, W. H. Smith, D. Taylor, P. Verwilligen, C. Vuosalo, N. Woods, CMS Collaboration

**Affiliations:** Yerevan Physics Institute, Yerevan, Armenia; Institut für Hochenergiephysik der OeAW, Vienna, Austria; National Centre for Particle and High Energy Physics, Minsk, Belarus; Universiteit Antwerpen, Antwerp, Belgium; Vrije Universiteit Brussel, Brussels, Belgium; Université Libre de Bruxelles, Brussels, Belgium; Ghent University, Ghent, Belgium; Université Catholique de Louvain, Louvain-la-Neuve, Belgium; Université de Mons, Mons, Belgium; Centro Brasileiro de Pesquisas Fisicas, Rio de Janeiro, Brazil; Universidade do Estado do Rio de Janeiro, Rio de Janeiro, Brazil; Universidade Estadual Paulista, Universidade Federal do ABC, São Paulo, Brazil; Institute for Nuclear Research and Nuclear Energy, Sofia, Bulgaria; University of Sofia, Sofia, Bulgaria; Institute of High Energy Physics, Beijing, China; State Key Laboratory of Nuclear Physics and Technology, Peking University, Beijing, China; Universidad de Los Andes, Bogotá, Colombia; Faculty of Electrical Engineering, Mechanical Engineering and Naval Architecture, University of Split, Split, Croatia; Faculty of Science, University of Split, Split, Croatia; Institute Rudjer Boskovic, Zagreb, Croatia; University of Cyprus, Nicosia, Cyprus; Charles University, Prague, Czech Republic; Academy of Scientific Research and Technology of the Arab Republic of Egypt, Egyptian Network of High Energy Physics, Cairo, Egypt; National Institute of Chemical Physics and Biophysics, Tallinn, Estonia; Department of Physics, University of Helsinki, Helsinki, Finland; Helsinki Institute of Physics, Helsinki, Finland; Lappeenranta University of Technology, Lappeenranta, Finland; DSM/IRFU, CEA/Saclay, Gif-sur-Yvette, France; Laboratoire Leprince-Ringuet, Ecole Polytechnique, IN2P3-CNRS, Palaiseau, France; Institut Pluridisciplinaire Hubert Curien, Université de Strasbourg, Université de Haute Alsace Mulhouse, CNRS/IN2P3, Strasbourg, France; Centre de Calcul de l’Institut National de Physique Nucleaire et de Physique des Particules, CNRS/IN2P3, Villeurbanne, France; Institut de Physique Nucléaire de Lyon, Université de Lyon, Université Claude Bernard Lyon 1, CNRS-IN2P3, Villeurbanne, France; Institute of High Energy Physics and Informatization, Tbilisi State University, Tbilisi, Georgia; I. Physikalisches Institut, RWTH Aachen University, Aachen, Germany; III. Physikalisches Institut A, RWTH Aachen University, Aachen, Germany; III. Physikalisches Institut B, RWTH Aachen University, Aachen, Germany; Deutsches Elektronen-Synchrotron, Hamburg, Germany; University of Hamburg, Hamburg, Germany; Institut für Experimentelle Kernphysik, Karlsruhe, Germany; Institute of Nuclear and Particle Physics (INPP), NCSR Demokritos, Aghia Paraskevi, Greece; University of Athens, Athens, Greece; University of Ioánnina, Ioannina, Greece; Wigner Research Centre for Physics, Budapest, Hungary; Institute of Nuclear Research ATOMKI, Debrecen, Hungary; University of Debrecen, Debrecen, Hungary; National Institute of Science Education and Research, Bhubaneswar, India; Panjab University, Chandigarh, India; University of Delhi, Delhi, India; Saha Institute of Nuclear Physics, Kolkata, India; Bhabha Atomic Research Centre, Mumbai, India; Tata Institute of Fundamental Research, Mumbai, India; Institute for Research in Fundamental Sciences (IPM), Tehran, Iran; University College Dublin, Dublin, Ireland; INFN Sezione di Bari, Università di Bari, Politecnico di Bari, Bari, Italy; INFN Sezione di Bologna, Università di Bologna, Bologna, Italy; INFN Sezione di Catania, Università di Catania, CSFNSM, Catania, Italy; INFN Sezione di Firenze, Università di Firenze, Florence, Italy; INFN Laboratori Nazionali di Frascati, Frascati, Italy; INFN Sezione di Genova, Università di Genova, Genoa, Italy; INFN Sezione di Milano-Bicocca, Università di Milano-Bicocca, Milan, Italy; INFN Sezione di Napoli, Università di Napoli ‘Federico II’, Università della Basilicata (Potenza), Università G. Marconi (Roma), Naples, Italy; INFN Sezione di Padova, Università di Padova, Università di Trento (Trento), Padua, Italy; INFN Sezione di Pavia, Università di Pavia, Pavia, Italy; INFN Sezione di Perugia, Università di Perugia, Perugia, Italy; INFN Sezione di Pisa, Università di Pisa, Scuola Normale Superiore di Pisa, Pisa, Italy; INFN Sezione di Roma, Università di Roma, Rome, Italy; INFN Sezione di Torino, Università di Torino, Università del Piemonte Orientale (Novara), Turin, Italy; INFN Sezione di Trieste, Università di Trieste, Trieste, Italy; Kangwon National University, Chunchon, Korea; Kyungpook National University, Taegu, Korea; Chonbuk National University, Chonju, Korea; Chonnam National University, Institute for Universe and Elementary Particles, Kwangju, Korea; Korea University, Seoul, Korea; University of Seoul, Seoul, Korea; Sungkyunkwan University, Suwon, Korea; Vilnius University, Vilnius, Lithuania; National Centre for Particle Physics, Universiti Malaya, Kuala Lumpur, Malaysia; Centro de Investigacion y de Estudios Avanzados del IPN, Mexico City, Mexico; Universidad Iberoamericana, Mexico City, Mexico; Benemerita Universidad Autonoma de Puebla, Puebla, Mexico; Universidad Autónoma de San Luis Potosí, San Luis Potosí, Mexico; University of Auckland, Auckland, New Zealand; University of Canterbury, Christchurch, New Zealand; National Centre for Physics, Quaid-I-Azam University, Islamabad, Pakistan; National Centre for Nuclear Research, Swierk, Poland; Institute of Experimental Physics, Faculty of Physics, University of Warsaw, Warsaw, Poland; Laboratório de Instrumentação e Física Experimental de Partículas, Lisbon, Portugal; Joint Institute for Nuclear Research, Dubna, Russia; Petersburg Nuclear Physics Institute, Gatchina, St. Petersburg, Russia; Institute for Nuclear Research, Moscow, Russia; Institute for Theoretical and Experimental Physics, Moscow, Russia; P. N. Lebedev Physical Institute, Moscow, Russia; Skobeltsyn Institute of Nuclear Physics, Lomonosov Moscow State University, Moscow, Russia; State Research Center of Russian Federation, Institute for High Energy Physics, Protvino, Russia; Faculty of Physics and Vinca Institute of Nuclear Sciences, University of Belgrade, Belgrade, Serbia; Centro de Investigaciones Energéticas Medioambientales y Tecnológicas (CIEMAT), Madrid, Spain; Universidad Autónoma de Madrid, Madrid, Spain; Universidad de Oviedo, Oviedo, Spain; Instituto de Física de Cantabria (IFCA), CSIC-Universidad de Cantabria, Santander, Spain; CERN, European Organization for Nuclear Research, Geneva, Switzerland; Paul Scherrer Institut, Villigen, Switzerland; Institute for Particle Physics, ETH Zurich, Zurich, Switzerland; Universität Zürich, Zurich, Switzerland; National Central University, Chung-Li, Taiwan; National Taiwan University (NTU), Taipei, Taiwan; Department of Physics, Faculty of Science, Chulalongkorn University, Bangkok, Thailand; Cukurova University, Adana, Turkey; Physics Department, Middle East Technical University, Ankara, Turkey; Bogazici University, Istanbul, Turkey; Istanbul Technical University, Istanbul, Turkey; National Scientific Center, Kharkov Institute of Physics and Technology, Kharkov, Ukraine; University of Bristol, Bristol, UK; Rutherford Appleton Laboratory, Didcot, UK; Imperial College, London, UK; Brunel University, Uxbridge, UK; Baylor University, Waco, USA; The University of Alabama, Tuscaloosa, USA; Boston University, Boston, USA; Brown University, Providence, USA; University of California, Davis, USA; University of California, Los Angeles, USA; University of California, Riverside, Riverside, USA; University of California, San Diego, La Jolla, USA; University of California, Santa Barbara, Santa Barbara, USA; California Institute of Technology, Pasadena, USA; Carnegie Mellon University, Pittsburgh, USA; University of Colorado at Boulder, Boulder, USA; Cornell University, Ithaca, USA; Fairfield University, Fairfield, USA; Fermi National Accelerator Laboratory, Batavia, USA; University of Florida, Gainesville, USA; Florida International University, Miami, USA; Florida State University, Tallahassee, USA; Florida Institute of Technology, Melbourne, USA; University of Illinois at Chicago (UIC), Chicago, USA; The University of Iowa, Iowa City, USA; Johns Hopkins University, Baltimore, USA; The University of Kansas, Lawrence, USA; Kansas State University, Manhattan, USA; Lawrence Livermore National Laboratory, Livermore, USA; University of Maryland, College Park, USA; Massachusetts Institute of Technology, Cambridge, USA; University of Minnesota, Minneapolis, USA; University of Mississippi, Oxford, USA; University of Nebraska-Lincoln, Lincoln, USA; State University of New York at Buffalo, Buffalo, USA; Northeastern University, Boston, USA; Northwestern University, Evanston, USA; University of Notre Dame, Notre Dame, USA; The Ohio State University, Columbus, USA; Princeton University, Princeton, USA; University of Puerto Rico, Mayagüez, USA; Purdue University, West Lafayette, USA; Purdue University Calumet, Hammond, USA; Rice University, Houston, USA; University of Rochester, Rochester, USA; The Rockefeller University, New York, USA; Rutgers, The State University of New Jersey, Piscataway, USA; University of Tennessee, Knoxville, USA; Texas A&M University, College Station, USA; Texas Tech University, Lubbock, USA; Vanderbilt University, Nashville, USA; University of Virginia, Charlottesville, USA; Wayne State University, Detroit, USA; University of Wisconsin, Madison, USA; CERN, 1211 Geneva 23, Switzerland

## Abstract

The inclusive jet cross section for proton–proton collisions at a centre-of-mass energy of 7$\,\text {TeV}$ was measured by the CMS Collaboration at the LHC with data corresponding to an integrated luminosity of 5.0$\,\text {fb}^{-1}$. The measurement covers a phase space up to 2$\,\text {TeV}$ in jet transverse momentum and 2.5 in absolute jet rapidity. The statistical precision of these data leads to stringent constraints on the parton distribution functions of the proton. The data provide important input for the gluon density at high fractions of the proton momentum and for the strong coupling constant at large energy scales. Using predictions from perturbative quantum chromodynamics at next-to-leading order, complemented with electroweak corrections, the constraining power of these data is investigated and the strong coupling constant at the Z boson mass $M_{\mathrm{Z}}$ is determined to be $\alpha _S(M_{\mathrm{Z}}) = 0.1185\pm 0.0019\,(\text {exp})^{+0.0060}_{-0.0037}\,\text {(theo)}$, which is in agreement with the world average.

## Introduction

Collimated streams of particles, conventionally called jets, are abundantly produced in highly energetic proton–proton collisions at the LHC. At high transverse momenta $$p_{\mathrm {T}}$$ these collisions are described by quantum chromodynamics (QCD) using perturbative techniques (pQCD). Indispensable ingredients for QCD predictions of cross sections in $$\mathrm {p}$$$$\mathrm {p}$$ collisions are the proton structure, expressed in terms of parton distribution functions (PDFs), and the strong coupling constant $$\alpha _S$$, which is a fundamental parameter of QCD. The PDFs and $$\alpha _S$$ both depend on the relevant energy scale *Q* of the scattering process, which is identified with the jet $$p_{\mathrm {T}}$$ for the reactions considered in this report. In addition, the PDFs, defined for each type of parton, depend on the fractional momentum *x* of the proton carried by the parton.

The large cross section for jet production at the LHC and the unprecedented experimental precision of the jet measurements allow stringent tests of QCD. In this study, the theory is confronted with data in previously inaccessible phase space regions of *Q* and *x*. When jet production cross sections are combined with inclusive data from deep-inelastic scattering (DIS), the gluon PDF for $x \gtrsim 0.01$ can be constrained and $\alpha _S(M_{\mathrm{Z}})$ can be determined. In the present analysis, this is demonstrated by means of the CMS measurement of inclusive jet production [1]. The data, collected in 2011 and corresponding to an integrated luminosity of 5.0$\,\text {fb}^{-1}$, extend the accessible phase space in jet $p_{\mathrm {T}}$ up to 2$\,\text {TeV}$, and range up to $|y | = 2.5$ in absolute jet rapidity. A PDF study using inclusive jet measurements by the ATLAS Collaboration is described in Ref. [2].

This paper is divided into six parts. Sect. 2 presents an overview of the CMS detector and of the measurement, published in Ref. [1], and proposes a modified treatment of correlations in the experimental uncertainties. Theoretical ingredients are introduced in Sect. 3. Section 4 is dedicated to the determination of $\alpha _S$ at the scale of the ${\mathrm{Z}}$-boson mass $M_{\mathrm{Z}}$, and in Sect. 5 the influence of the jet data on the PDFs is discussed. A summary is presented in Sect. 6.

## The inclusive jet cross section

### Overview of the CMS detector and of the measurement

The central feature of the CMS detector is a superconducting solenoid of 6$\,\text {m}$ internal diameter, providing a magnetic field of 3.8$\,\text {T}$. Within the superconducting solenoid volume are a silicon pixel and strip tracker, a lead tungstate crystal electromagnetic calorimeter (ECAL), and a brass/scintillator hadron calorimeter, each composed of a barrel and two endcap sections. Muons are measured in gas-ionisation detectors embedded in the steel flux-return yoke outside the solenoid. Extensive forward calorimetry (HF) complements the coverage provided by the barrel and endcap detectors. A more detailed description of the CMS detector, together with a definition of the coordinate system used and the relevant kinematic variables, can be found in Ref. [3].

Jets are reconstructed with a size parameter of $R=0.7$ using the collinear- and infrared-safe anti-$k_{\mathrm {T}}$ clustering algorithm [4] as implemented in the FastJet package [5]. The published measurements of the cross sections were corrected for detector effects, and include statistical and systematic experimental uncertainties as well as bin-to-bin correlations for each type of uncertainty. A complete description of the measurement can be found in Ref. [1].

The double-differential inclusive jet cross section investigated in the following is derived from observed inclusive jet yields via

1 $$\begin{aligned} \frac{{\mathrm{d}}^2\sigma }{{\mathrm{d}}p_{\mathrm {T}} \,{\mathrm{d}}y} = \frac{1}{\epsilon \cdot \mathcal {L}_{\text {int}}} \frac{N_\text {jets}}{\Delta p_{\mathrm {T}} \,\left( 2\cdot \Delta |y | \right) }, \end{aligned}$$

where $N_\text {jets}$ is the number of jets in the specific kinematic range (bin), $\mathcal {L}_{\text {int}}$ is the integrated luminosity, $\epsilon$ is the product of trigger and event selection efficiencies, and $\Delta p_{\mathrm {T}}$ and $\Delta |y |$ are the bin widths in $p_{\mathrm {T}}$ and $|y |$. The factor of two reflects the folding of the distributions around $y=0$.

### Experimental uncertainties

The inclusive jet cross section is measured in five equally sized bins of $\Delta |y | = 0.5$ up to an absolute rapidity of $|y | = 2.5$. The inner three regions roughly correspond to the barrel part of the detector, the outer two to the endcaps. Tracker coverage extends up to $|y | = 2.4$. The minimum $p_{\mathrm {T}}$ imposed on any jet is 114$\,\text {GeV}$. The binning in jet $p_{\mathrm {T}}$ follows the jet $p_{\mathrm {T}}$ resolution of the central detector and changes with $p_{\mathrm {T}}$. The upper reach in $p_{\mathrm {T}}$ is given by the available data and decreases with $|y |$.

Four categories [1] of experimental uncertainties are defined: the jet energy scale (JES), the luminosity, the corrections for detector response and resolution, and all remaining uncorrelated effects.

The JES is the dominant source of systematic uncertainty, because a small shift in the measured $p_{\mathrm {T}}$ translates into a large uncertainty in the steeply falling jet $p_{\mathrm {T}}$ spectrum and hence in the cross section for any given value of $p_{\mathrm {T}}$. The JES uncertainty is parameterized in terms of jet $p_{\mathrm {T}}$ and pseudorapidity $\eta = -\ln \tan (\theta /2)$ and amounts to 1–2 % [6], which translates into a 5–25 % uncertainty in the cross section. Because of its particular importance for this analysis, more details are given in Sect. 2.3.

The uncertainty in the integrated luminosity is 2.2 % [7] and translates into a normalisation uncertainty that is fully correlated across $|y |$ and $p_{\mathrm {T}}$.

The effect of the jet energy resolution (JER) is corrected for using the D’Agostini method [8] as implemented in the RooUnfold package [9]. The uncertainty due to the unfolding comprises the effects of an imprecise knowledge of the JER, of residual differences between data and the Monte Carlo (MC) modelling of detector response, and of the unfolding technique applied. The total unfolding uncertainty, which is fully correlated across $\eta$ and $p_{\mathrm {T}}$, is 3–4 %. Additionally, the statistical uncertainties are propagated through the unfolding procedure, thereby providing the correlations between the statistical uncertainties of the unfolded measurement. A statistical covariance matrix must be used to take this into account.

Remaining effects are collected into an uncorrelated uncertainty of $\approx$1 %.

### Uncertainties in JES

The procedure to calibrate jet energies in CMS and ways to estimate JES uncertainties are described in Ref. [10]. To use CMS data in fits of PDFs or $\alpha _S(M_{\mathrm{Z}})$, it is essential to account for the correlations in these uncertainties among different regions of the detector. The treatment of correlations uses 16 mutually uncorrelated sources as in Ref. [1]. Within each source, the uncertainties are fully correlated in $p_{\mathrm {T}}$ and $\eta$. Any change in the jet energy calibration (JEC) is described through a linear combination of sources, where each source is assumed to have a Gaussian probability density with a zero mean and a root-mean-square of unity. In this way, the uncertainty correlations are encoded in a fashion similar to that provided for PDF uncertainties using the Hessian method [11]. The total uncertainty is defined through the quadratic sum of all uncertainties. The full list of sources together with their brief descriptions can be found in Appendix A.

The JES uncertainties can be classified into four broad categories: absolute energy scale as a function of $p_{\mathrm {T}}$, jet flavour dependent differences, relative calibration of JES as a function of $\eta$, and the effects of multiple proton interactions in the same or adjacent beam crossings (pileup). The absolute scale is a single fixed number such that the corresponding uncertainty is fully correlated across $p_{\mathrm {T}}$ and $\eta$. Using photon $+$ jet and *Z* $+$ jet data, the JES can be constrained directly in the jet $p_{\mathrm {T}}$ range 30–600$\,\text {GeV}$. The response at larger and smaller $p_{\mathrm {T}}$ is extrapolated through MC simulation. Extra uncertainties are assigned to this extrapolation based on the differences between MC event generators and the single-particle response of the detector. The absolute calibration is the most relevant uncertainty in jet analyses at large $p_{\mathrm {T}}$.

The categories involving jet flavour dependence and pileup effects are important mainly at small $p_{\mathrm {T}}$ and have relatively little impact for the phase space considered in this report.

The third category parameterizes $\eta$-dependent changes in relative JES. The measurement uncertainties within different detector regions are strongly correlated, and thus the $\eta$-dependent sources are only provided for wide regions: barrel, endcap with upstream tracking, endcap without upstream tracking, and the HF calorimeter. In principle, the $\eta$-dependent effects can also have a $p_{\mathrm {T}}$ dependence. Based on systematic studies on data and simulated events, which indicate that the $p_{\mathrm {T}}$ and $\eta$ dependence of the uncertainties factorise to a good approximation, this is omitted from the initial calibration procedure. However, experiences with the calibration of data collected in 2012 and with fits of $\alpha _S(M_{\mathrm{Z}})$ reported in Sect. 4 show that this is too strong an assumption. Applying the uncertainties and correlations in a fit of $\alpha _S(M_{\mathrm{Z}})$ to the inclusive jet data separately for each bin in $|y |$ leads to results with values of $\alpha _S(M_{\mathrm{Z}})$ that scatter around a central value. Performing the same fit taking all $|y |$ bins together and assuming 100 % correlation in $|y |$ within the JES uncertainty sources results in a bad fit quality (high $\chi ^2$ per number of degrees of freedom $n_\mathrm {dof}$) and a value of $\alpha _S(M_{\mathrm{Z}})$ that is significantly higher than any value observed for an individual bin in $|y |$. Changing the correlation in the JES uncertainty from 0 to 100 % produces a steep rise in $\chi ^2/n_\mathrm {dof}$, and influences the fitted value of $\alpha _S(M_{\mathrm{Z}})$ for correlations near 90 %, indicating an assumption on the correlations in $|y |$ that is too strong. The technique of nuisance parameters, as described in Sect. 5.2.2, helped in the analysis of this issue.

To implement the additional $\eta$-decorrelation induced by the $p_{\mathrm {T}}$-dependence in the $\eta$-dependent JEC introduced for the calibration of 2012 data, the source from the single-particle response JEC2, which accounts for extrapolation uncertainties at large $p_{\mathrm {T}}$ as discussed in Appendix A, is decorrelated versus $\eta$ as follows:

1. in the barrel region ($|y | < 1.5$), the correlation of the single-particle response source among the three bins in $|y |$ is set to 50 %,
2. in the endcap region ($1.5 \le |y | < 2.5$), the correlation of the single-particle response source between the two bins in $|y |$ is kept at 100 %,
3. there is no correlation of the single-particle response source between the two detector regions of $|y | < 1.5$ and $1.5 \le |y | < 2.5$.

The additional freedom of $p_{\mathrm {T}}$-dependent corrections versus $\eta$ hence leads to a modification of the previously assumed full correlation between all $\eta$ regions to a reduced estimate of 50 % correlation of JEC2 within the barrel region, which always contains the tag jet of the dijet balance method [10]. In addition, the JEC2 corrections are estimated to be uncorrelated between the barrel and endcap regions of the detector because of respective separate $p_{\mathrm {T}}$-dependences of these corrections.

**Table 1:** The PDF sets used in comparisons to the data together with the evolution order (Evol.), the corresponding number of active flavours $N_f$, the assumed masses $M_{\mathrm{t}}$ and $M_{\mathrm{Z}}$ of the top quark and the ${\mathrm{Z}}$ boson, respectively, the default values of $\alpha _S(M_{\mathrm{Z}})$, and the range in $\alpha _S(M_{\mathrm{Z}})$ variation available for fits. For CT10 the updated versions of 2012 are taken

Base set	Refs.	Evol.	$N_f$	$M_{\mathrm{t}}$ ($\text {GeV}$ )	$M_{\mathrm{Z}}$ ($\text {GeV}$ )	$\alpha _S(M_{\mathrm{Z}})$	$\alpha _S(M_{\mathrm{Z}})$ range
ABM11	[17]	NLO	5	180	91.174	0.1180	0.110–0.130
ABM11	[17]	NNLO	5	180	91.174	0.1134	0.104–0.120
CT10	[18]	NLO	${\le }5$	172	91.188	0.1180	0.112–0.127
CT10	[18]	NNLO	${\le }5$	172	91.188	0.1180	0.110–0.130
HERAPDF1.5	[19]	NLO	${\le }5$	180	91.187	0.1176	0.114–0.122
HERAPDF1.5	[19]	NNLO	${\le }5$	180	91.187	0.1176	0.114–0.122
MSTW2008	[20, 21]	NLO	${\le }5$	$10^{10}$	91.1876	0.1202	0.110–0.130
MSTW2008	[20, 21]	NNLO	${\le }5$	$10^{10}$	91.1876	0.1171	0.107–0.127
NNPDF2.1	[22]	NLO	${\le }6$	175	91.2	0.1190	0.114–0.124
NNPDF2.1	[22]	NNLO	${\le }6$	175	91.2	0.1190	0.114–0.124

Technically, this can be achieved by splitting the single-particle response source into five parts (JEC2a–e), as shown in Table 8. Each of these sources is a duplicate of the original single-particle response source that is set to zero outside the respective ranges of $|y | < 1.5$, $1.5 \le |y | < 2.5$, $|y | < 0.5$, $0.5 \le |y | < 1.0$, and $1.0 \le |y | < 1.5$, such that the original full correlation of

2 $$\begin{aligned} \mathrm {corr}_\mathrm {JEC2,old} = \begin{pmatrix} 1 &{}\quad 1 &{}\quad 1 &{}\quad 1 &{}\quad 1\\ 1 &{}\quad 1 &{}\quad 1 &{}\quad 1 &{}\quad 1\\ 1 &{}\quad 1 &{}\quad 1 &{}\quad 1 &{}\quad 1\\ 1 &{}\quad 1 &{}\quad 1 &{}\quad 1 &{}\quad 1\\ 1 &{}\quad 1 &{}\quad 1 &{}\quad 1 &{}\quad 1\\ \end{pmatrix} \end{aligned}$$

is replaced by the partially uncorrelated version of

3 $$\begin{aligned} \mathrm {corr}_\mathrm {JEC2,new} = \begin{pmatrix} 1 &{}\quad 0.5 &{}\quad 0.5 &{}\quad 0 &{}\quad 0\\ 0.5 &{}\quad 1 &{}\quad 0.5 &{}\quad 0 &{}\quad 0\\ 0.5 &{}\quad 0.5 &{}\quad 1 &{}\quad 0 &{}\quad 0\\ 0 &{}\quad 0 &{}\quad 0 &{}\quad 1 &{}\quad 1\\ 0 &{}\quad 0 &{}\quad 0 &{}\quad 1 &{}\quad 1\\ \end{pmatrix}, \end{aligned}$$

which is more accurate as justified by studies based on 2012 data. For the proper normalisation of the five new correlated sources, normalisation factors of $1/\sqrt{2}$ (JEC2a, JEC2c–JEC2f) and 1 (JEC2b) must be applied. With these factors, the sum of the five sources reproduces the original uncertainty for each $|y |$, while the additional freedom gives the estimated level of correlation among the $|y |$ regions.

All results presented in this paper are based on this improved treatment of the correlation of JES uncertainties. While some decorrelation of these uncertainties versus $\eta$ is important for the fits of $\alpha _S(M_{\mathrm{Z}})$ described in Sect. 4, the exact size of the estimated decorrelation is not. Varying the assumptions according to Eq. (3) from 50 % to 20 or 80 % in the barrel region, from 100 to 80 % in the endcap region, or from 0 to 20 % between the barrel and endcap region leads to changes in the fitted value of $\alpha _S(M_{\mathrm{Z}})$ that are negligible with respect to other experimental uncertainties.

## Theoretical ingredients

The theoretical predictions for the inclusive jet cross section comprise a next-to-leading order (NLO) pQCD calculation with electroweak corrections (EW) [12, 13]. They are complemented by a nonperturbative (NP) factor that corrects for multiple-parton interactions (MPI) and hadronization (HAD) effects. Parton shower (PS) corrections, derived from NLO predictions with matched parton showers, are tested in an additional study in Sect. 4.3, but are not applied to the main result.

### Fixed-order prediction in perturbative QCD

The same NLO prediction as in Ref. [1] is used, i.e. the calculations are based on the parton-level program NLOJet++ version 4.1.3 [14, 15] and are performed within the fastNLO framework version 2.1 [16]. The renormalization and factorisation scales, $\mu _r$ and $\mu _f$ respectively, are identified with the individual jet $p_{\mathrm {T}}$. The number of active (massless) flavours $N_f$ in NLOJet++ has been set to five.

Five sets of PDFs are available for a series of values of $\alpha _S(M_{\mathrm{Z}})$, which is a requisite for a determination of $\alpha _S(M_{\mathrm{Z}})$ from data. For an overview, these PDF sets are listed in Table 1 together with the respective references. The ABM11 PDF set employs a fixed-flavour number scheme with five active flavours, while the other PDF sets use a variable-flavour number scheme with a maximum of five flavours, $N_{f,\mathrm {max}} = 5$, except for NNPDF2.1 which has $N_{f,\mathrm {max}} = 6$. All sets exist at next-to-leading and next-to-next-to-leading evolution order. The PDF uncertainties are provided at 68.3 % confidence level (CL) except for CT10, which provides uncertainties at $90\,\%$ CL. For a uniform treatment of all PDFs, the CT10 uncertainties are downscaled by a factor of $\sqrt{2}{{\mathrm{erf}}}^{-1}{(0.9)} \approx 1.645$.

The electroweak corrections to the hard-scattering cross section have been computed with the CT10-NLO PDF set for a fixed number of five flavours and with the $p_{\mathrm {T}}$ of the leading jet, $p_{\mathrm {T,max}}$, as scale choice for $\mu _r$ and $\mu _f$ instead of the $p_{\mathrm {T}}$ of each jet. At high jet $p_{\mathrm {T}}$ and central rapidity, where the electroweak effects become sizeable, NLO calculations with either of the two scale settings differ by less than one percent. Given the small impact of the electroweak corrections on the final results in Sects. 4 and 5, no uncertainty on their size has been assigned.

### Theoretical prediction from MC simulations including parton showers and nonperturbative effects

The most precise theoretical predictions for jet measurements are usually achieved in fixed-order pQCD, but are available at parton level only. Data that have been corrected for detector effects, however, refer to measurable particles, i.e. to colour-neutral particles with mean decay lengths such that $c\tau >10\,\text {mm}$. Two complications arise when comparing fixed-order perturbation theory to these measurements: emissions of additional partons close in phase space, which are not sufficiently accounted for in low-order approximations, and effects that cannot be treated by perturbative methods. The first problem is addressed by the parton shower concept [23–25] within pQCD, where multiple parton radiation close in phase space is taken into account through an all-orders approximation of the dominant terms including coherence effects. Avoiding double counting, these parton showers are combined with leading-order (LO) calculations in MC event generators, such as pythia  [26] and herwig++ [27].

The second issue concerns NP corrections, which comprise supplementary parton-parton scatters within the same colliding protons, i.e. MPI, and the hadronization process including particle decays. The MPI [28, 29] model for additional soft-particle production, which is detected as part of the underlying event, is implemented in pythia as well as herwig++. Hadronization describes the transition phase from coloured partons to colour-neutral particles, where perturbative methods are no longer applicable. Two models for hadronization are in common use, the Lund string fragmentation [30–32] that is used in pythia, and the cluster fragmentation [33] that has been adopted by herwig++.

Beyond LO combining fixed-order predictions with parton showers, MPI, and hadronization models is much more complicated. Potential double counting of terms in the perturbative expansion and the PS has to be avoided. In recent years programs have become available for dijet production at NLO that can be matched to PS MC event generators. In the following, one such program, the powheg package [34, 35] will be used for comparisons with dijet events [36] to the LO MC event generators.

### NP corrections from pythia6 and herwig++

For the comparison of theoretical predictions to the measurement reported in Ref. [1], the NP correction was derived as usual [37] from the average prediction of two LO MC event generators and more specifically from pythia version 6.4.22 tune Z2 and herwig++ version 2.4.2 with the default tune of version 2.3. Tune Z2 is identical to tune Z1 described in [38] except that Z2 employs the CTEQ6L1 [39] PDF set, while Z1 uses the CTEQ5L [40] PDF set. The NP correction factor can be defined for each bin in $p_{\mathrm {T}}$ and $|y |$ as

4 $$\begin{aligned} C _\mathrm {LO}^{\text {NP}} = \frac{\sigma _{\mathrm {LO+PS+HAD+MPI}}}{\sigma _{\mathrm {LO+PS}}}\, \end{aligned}$$

where $\sigma$ represents the inclusive jet cross section and the subscripts “LO+PS+HAD+MPI” and “LO+PS” indicate which steps of a general MC event generation procedure have been run, see also Refs. [37, 41]. The central value is calculated by taking the average of the two predictions from pythia6 and herwig++.

In applying these factors as corrections for NP effects to NLO theory predictions, it is assumed that the NP corrections are universal, i.e. they are similar for LO and NLO.

### NP and PS corrections from powheg$+$pythia6

Alternative corrections are derived, which use the powheg box revision 197 with the CT10-NLO PDF set for the hard subprocess at NLO plus the leading emission [42] complemented with the matched showering, MPI, and hadronization from pythia6 version 6.4.26. The NLO event generation within the powheg framework, and the showering and hadronization process performed by pythia6 are done in independent steps.

**Fig. 1:**
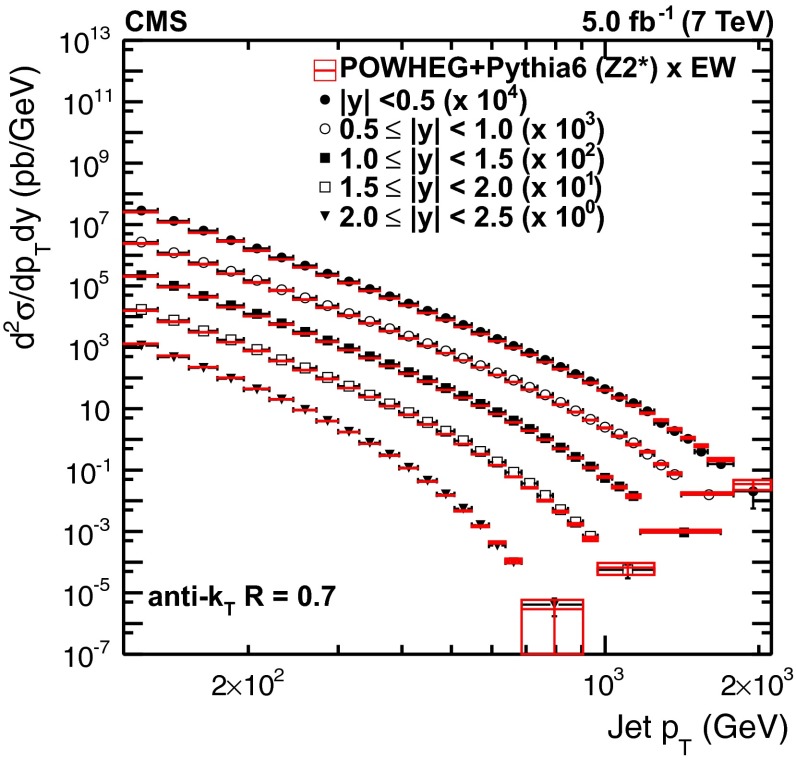
Measured inclusive jet cross section from Ref. [1] compared to the prediction by powheg $+$ pythia6 tune Z2* at particle level complemented with electroweak corrections. The *boxes* indicate the statistical uncertainty of the calculation

For illustration, Fig. 1 shows the comparison of the inclusive jet data with the powheg$+$pythia6 tune Z2* particle-level prediction complemented with electroweak corrections. The tune Z2* is derived from the earlier tune Z2, where the pythia6 parameters PARP(82) and PARP(90) that control the energy dependence of the MPI are retuned, yielding 1.921 and 0.227, respectively. The error boxes indicate statistical uncertainties. Ratio plots of this comparison for each separate region in $|y |$ can be found in Appendix B.

The corrections to NLO parton-level calculations that are derived this way consist of truly nonperturbative contributions, which are optionally complemented with parton shower effects. They are investigated separately in the following two sections. A previous investigation can be found in Ref. [43].

**Fig. 2:**
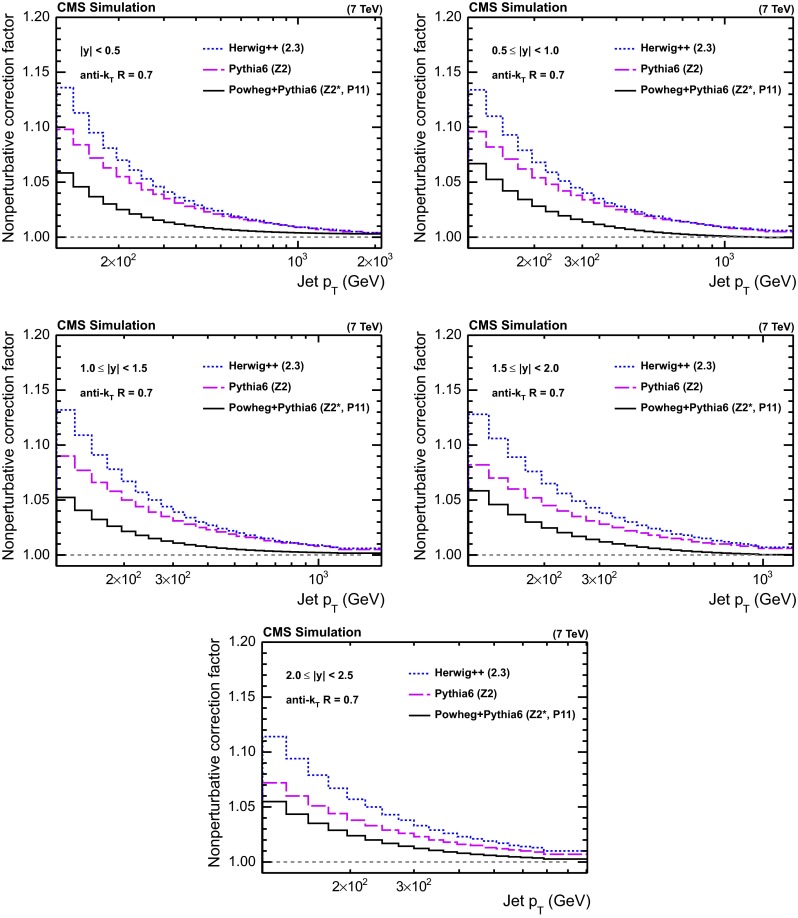
NP corrections for the five regions in $|y |$ as derived in Ref. [1], using pythia6 tune Z2 and herwig++ with the default tune of version 2.3, in comparison to corrections obtained from powheg using pythia6 for showering with the two underlying event tunes P11 and Z2*

**Fig. 3:**
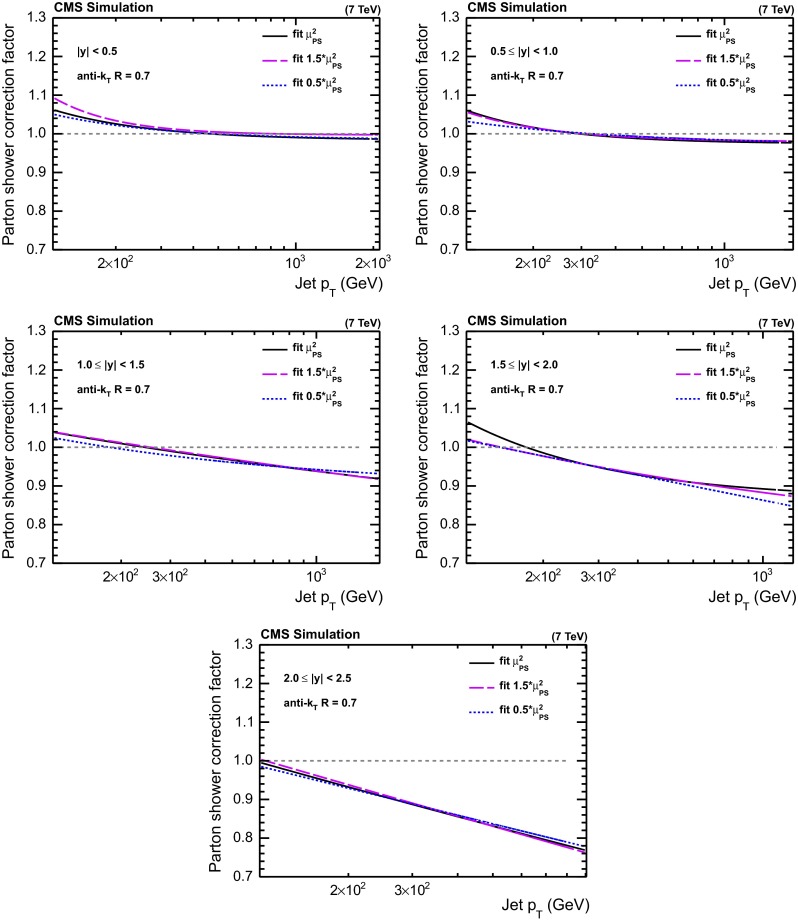
PS corrections for the five regions in $|y |$ obtained from powheg using pythia6 for showering for different upper scale limits of the parton shower evolution in pythia6 tune Z2*. The *curves* parameterize the correction factors as a function of the jet $p_{\mathrm {T}}$

#### NP corrections from powheg$+$pythia6

The NP corrections using a NLO prediction with a matched PS event generator can be defined analogously as in Eq. (4):

5 $$\begin{aligned} C_\mathrm {NLO}^{\text {NP}} = \frac{\sigma _{\text {NLO+PS+HAD+MPI}}}{\sigma _{\text {NLO+PS}}}, \end{aligned}$$

i.e. the numerator of this NP correction is defined by the inclusive cross section, where parton showers, hadronization, and multiparton interactions are turned on, while the inclusive cross section in the denominator does not include hadronization and multiparton interactions. A NLO calculation can then be corrected for NP effects as

6 $$\begin{aligned} \frac{{\mathrm{d}}^2 \sigma _\mathrm {theo}}{{\mathrm{d}}p_{\mathrm {T}} \, {\mathrm{d}}{}y} = \frac{{\mathrm{d}}^2 \sigma _\mathrm {NLO}}{{\mathrm{d}}p_{\mathrm {T}} \, {\mathrm{d}}{}y} \cdot C_\mathrm {NLO}^\mathrm {NP}. \end{aligned}$$

In contrast to the LO MC event generation with pythia6, the parameters of the NP and PS models, however, have not been retuned to data for the use with NLO $+$ PS predictions by powheg. Therefore two different underlying event tunes of pythia6 for LO $+$ PS predictions, P11 [44] and Z2*, are used. In both cases a parameterization using a functional form of $a_0 + a_1 / p_{\mathrm {T}} ^{a_2}$ is employed to smoothen statistical fluctuations. For $p_{\mathrm {T}} > 100\,\text {GeV}$ the difference in the NP correction factor between the two tunes is very small such that their average is taken as $C_\mathrm {NLO}^{\text {NP}}$.

Since procedures to estimate uncertainties inherent to the NLO $+$ PS matching procedure are not yet well established and proper tunes to data for powheg$+$pythia6 are lacking, the centre of the envelope given by the three curves from pythia6, herwig++, and the powheg$+$pythia6 average of tunes Z2* and P11 is adopted as the final NP correction for the central results in Sects. 4 and 5. Half the spread among these three predictions defines the uncertainty.

The NP correction, as defined for powheg$+$pythia6, is shown in Fig. 2 together with the original factors from pythia6 and herwig++, as a function of the jet $p_{\mathrm {T}}$ for five ranges in absolute rapidity $|y |$ of size 0.5 up to $|y | = 2.5$. The factors derived from both, LO $+$ PS and NLO $+$ PS MC event generators, are observed to decrease with increasing jet $p_{\mathrm {T}}$ and to approach unity at large $p_{\mathrm {T}}$. Within modelling uncertainties, the assumption of universal NP corrections that are similar for LO $+$ PS and NLO $+$ PS MC event generation holds approximately above a jet $p_{\mathrm {T}}$ of a few hundred $\,\text {GeV}$.

#### PS corrections from powheg$+$pythia6

Similarly to the NP correction of Eq. (5), a PS correction factor can be defined as the ratio of the differential cross section including PS effects divided by the NLO prediction, as given by powheg, i.e. including the leading emission:

7 $$\begin{aligned} C _\mathrm {NLO}^{\text {PS}} = \frac{\sigma _{\text {NLO+PS}}}{\sigma _{\text {NLO}}}. \end{aligned}$$

The combined correction for NP and PS effects can then be written as

8 $$\begin{aligned} \frac{{\mathrm{d}}^2 \sigma _\mathrm {theo}}{{\mathrm{d}}p_{\mathrm {T}} \, {\mathrm{d}}{}y} = \frac{{\mathrm{d}}^2 \sigma _\mathrm {NLO}}{{\mathrm{d}}p_{\mathrm {T}} \, {\mathrm{d}}{}y} \cdot C_\mathrm {NLO}^\mathrm {NP} \cdot C_\mathrm {NLO}^\mathrm {PS}. \end{aligned}$$

The PS corrections derived with powheg$+$pythia6 are presented in Fig. 3. They are significant at large $p_{\mathrm {T}}$, particularly at high rapidity, where the factors approach $-20$ %. However, the combination of powheg$+$pythia6 has never been tuned to data and the Z2* tune strictly is only valid for a LO $+$ PS tune with pythia6, but not with showers matched to powheg. Moreover, powheg employs the CT10-NLO PDF, while the Z2* tune requires the CTEQ6L1-LO PDF to be used for the showering part. Therefore, such PS corrections can be considered as only an illustrative test, as reported in Sect. 4.3.

The maximum parton virtuality allowed in the parton shower evolution, $\mu _\mathrm {PS}^2$, is varied by factors of 0.5 and 1.5 by changing the corresponding parameter PARP(67) in pythia6 from its default value of 4–2 and 6, respectively. The resulting changes in the PS factors are shown in Fig. 3. The powheg$+$pythia6 PS factors employed in an illustrative test later are determined as the average of the predictions from the two extreme scale limits. Again, a parameterization using a functional form of $a_0 + a_1 / p_{\mathrm {T}} ^{a_2}$ is employed to smoothen statistical fluctuations.

**Fig. 4:**
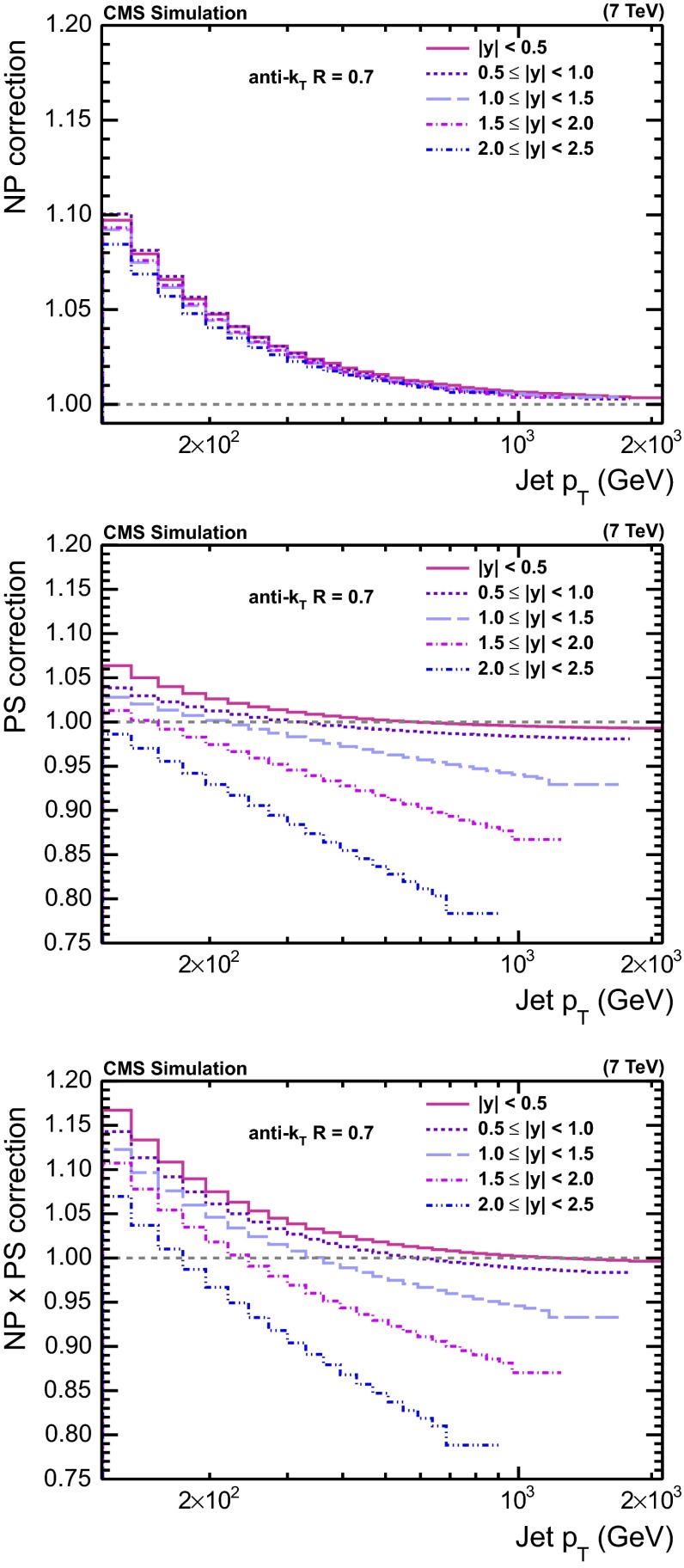
NP correction (*top*) obtained from the envelope of the predictions of pythia6 tune Z2, herwig++ tune 2.3, and powheg $+$ pythia6 with the tunes P11 and Z2*, PS correction (*middle*) obtained from the average of the predictions of powheg $+$ pythia6 tune Z2* with scale factor variation, and combined correction (*bottom*), defined as the product of the NP and PS correction, for the five regions in $|y |$

Finally, Fig. 4 presents an overview of the NP, PS, and combined corrections for all five ranges in $|y |$.

## Determination of the strong coupling constant

The measurement of the inclusive jet cross section [1], as described in Sect. 2, can be used to determine $\alpha _S(M_{\mathrm{Z}})$, where the proton structure in the form of PDFs is taken as a prerequisite. The necessary theoretical ingredients are specified in Sect. 3. The choice of PDF sets is restricted to global sets that fit data from different experiments, so that only the most precisely known gluon distributions are employed. Combined fits of $\alpha _S(M_{\mathrm{Z}})$ and the gluon content of the proton are investigated in Sect. 5.5.

In the following, the sensitivity of the inclusive jet cross section to $\alpha _S(M_{\mathrm{Z}})$ is demonstrated. Subsequently, the fitting procedure is given in detail before presenting the outcome of the various fits of $\alpha _S(M_{\mathrm{Z}})$.

### Sensitivity of the inclusive jet cross section to $\alpha _S(M_{\mathrm{Z}})$

**Fig. 5:**
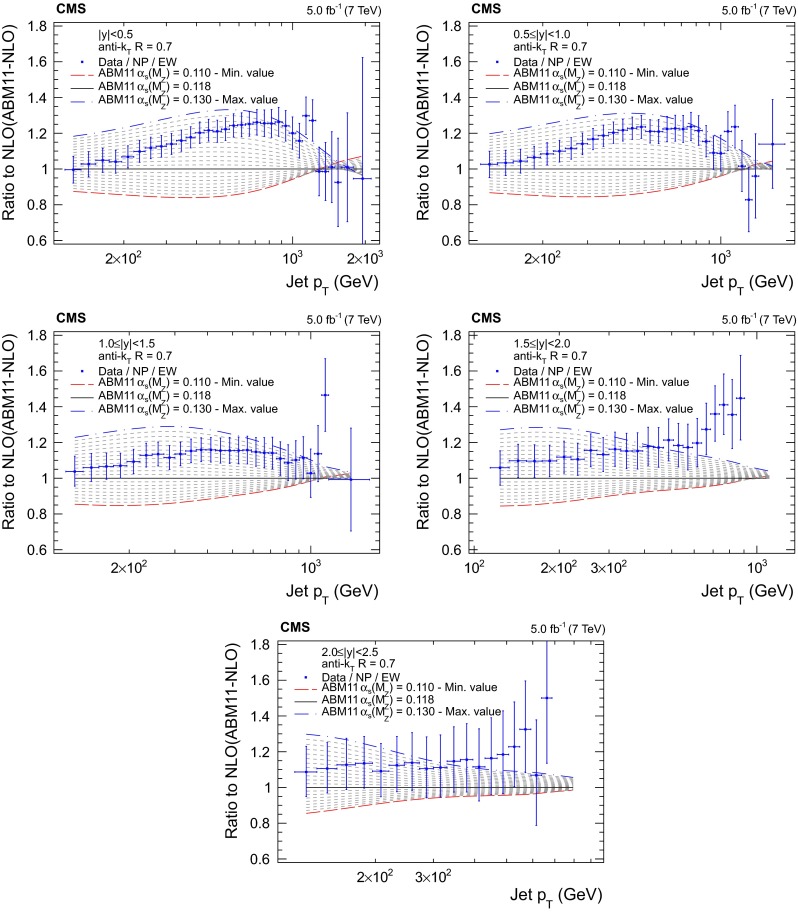
Ratio of the inclusive jet cross section to theoretical predictions using the ABM11-NLO PDF set for the five rapidity bins, where the $\alpha _S(M_{\mathrm{Z}})$ value is varied in the range 0.110–0.130 in steps of 0.001. The *error bars* correspond to the total uncertainty

**Fig. 6:**
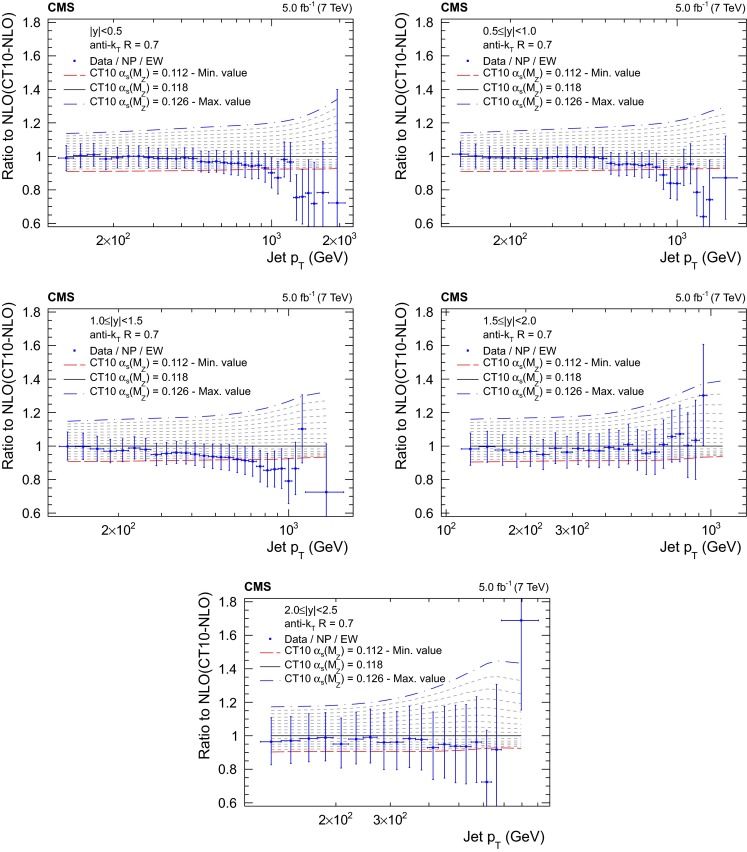
Ratio of the inclusive jet cross section to theoretical predictions using the CT10-NLO PDF set for the five rapidity bins, where the $\alpha _S(M_{\mathrm{Z}})$ value is varied in the range 0.112–0.126 in steps of 0.001. The *error bars* correspond to the total uncertainty

**Fig. 7:**
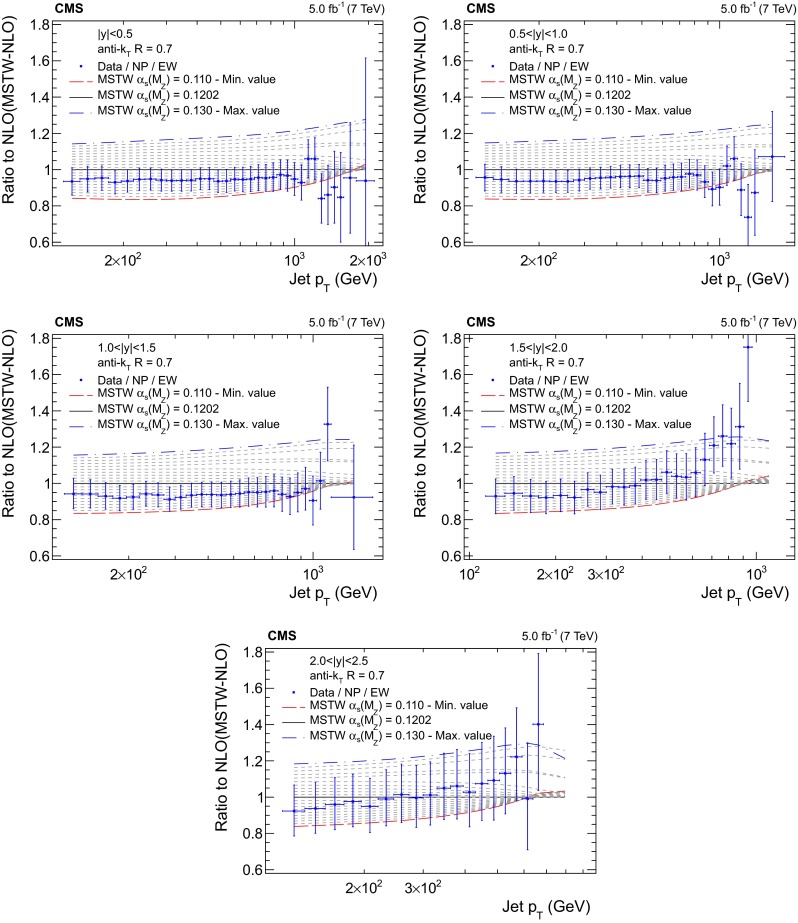
Ratio of the inclusive jet cross section to theoretical predictions using the MSTW2008-NLO PDF set for the five rapidity bins, where the $\alpha _S(M_{\mathrm{Z}})$ value is varied in the range 0.110–0.130 in steps of 0.001. The *error bars* correspond to the total uncertainty

**Fig. 8:**
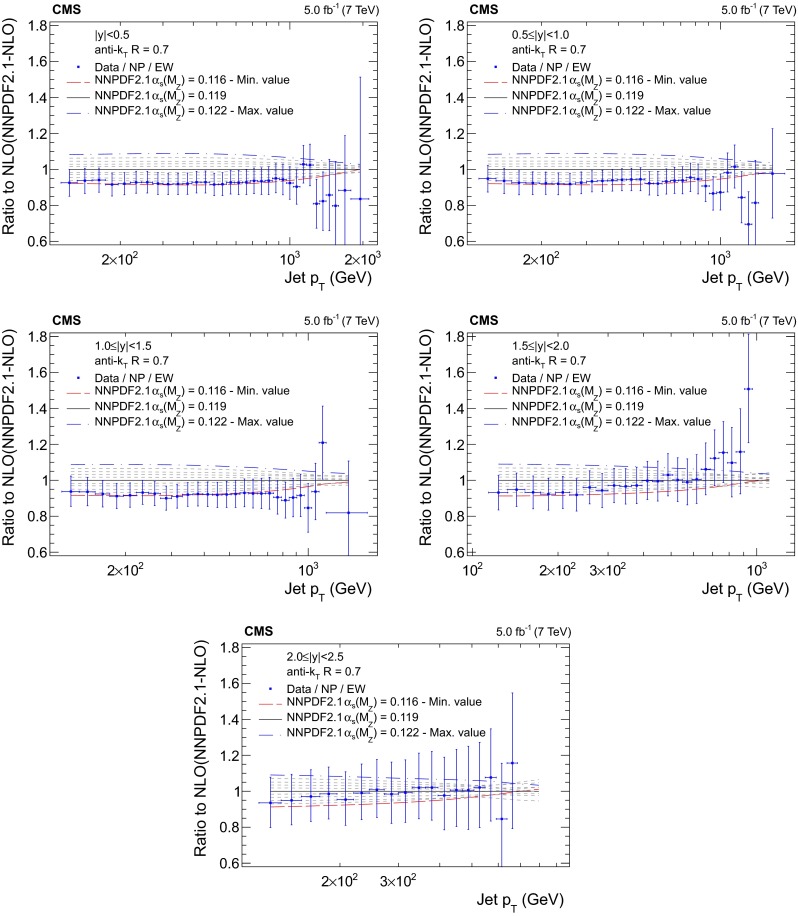
Ratio of the inclusive jet cross section to theoretical predictions using the NNPDF2.1-NLO PDF set for the five rapidity bins, where the $\alpha _S(M_{\mathrm{Z}})$ value is varied in the range 0.116–0.122 in steps of 0.001. The *error bars* correspond to the total uncertainty

Figures 5, 6, 7 and 8 present the ratio of data to the theoretical predictions for all variations in $\alpha _S(M_{\mathrm{Z}})$ available for the PDF sets ABM11, CT10, MSTW2008, and NNPDF2.1 at next-to-leading evolution order, as specified in Table 1. Except for the ABM11 PDF set, which leads to QCD predictions significantly different in shape to the measurement, all PDF sets give satisfactory theoretical descriptions of the data and a strong sensitivity to $\alpha _S(M_{\mathrm{Z}})$ is demonstrated. Because of the discrepancies, ABM11 is excluded from further investigations. The CT10-NLO PDF set is chosen for the main result on $\alpha _S(M_{\mathrm{Z}})$, because the value of $\alpha _S(M_{\mathrm{Z}})$ preferred by the CMS jet data is rather close to the default value of this PDF set. As crosschecks fits are performed with the NNPDF2.1-NLO and MSTW2008-NLO sets. The CT10-NNLO, NNPDF2.1-NNLO, and MSTW2008-NNLO PDF sets are employed for comparison.

### The fitting procedure

The value of $\alpha _S(M_{\mathrm{Z}})$ is determined by minimising the $\chi ^2$ between the *N* measurements $D_i$ and the theoretical predictions $T_i$. The $\chi ^2$ is defined as

9 $$\begin{aligned} \chi ^2 = \sum _{ij}^N (D_i - T_i) \mathrm {C}_{ij}^{-1} (D_j - T_j), \end{aligned}$$

where the covariance matrix $C_{ij}$ is composed of the following terms:

10 $$\begin{aligned} C= & {} {{\mathrm{cov}}}_\text {stat} + {{\mathrm{cov}}}_\text {uncor} + \left( \sum _\text {sources}{{\mathrm{cov}}}_\mathrm {JES}\right) + {{\mathrm{cov}}}_\text {unfolding} \nonumber \\&+ {{\mathrm{cov}}}_\text {lumi} + {{\mathrm{cov}}}_\mathrm {PDF}, \end{aligned}$$

and the terms in the sum represent

1. ${{\mathrm{cov}}}_\text {stat}$: statistical uncertainty including correlations induced through unfolding;
2. ${{\mathrm{cov}}}_\text {uncor}$: uncorrelated systematic uncertainty summing up small residual effects such as trigger and identification inefficiencies, time dependence of the jet $p_{\mathrm {T}}$ resolution, or the uncertainty on the trigger prescale factor;
3. ${{\mathrm{cov}}}_\mathrm {JES\,sources}$: systematic uncertainty for each JES uncertainty source;
4. ${{\mathrm{cov}}}_\text {unfolding}$: systematic uncertainty of the unfolding;
5. ${{\mathrm{cov}}}_\text {lumi}$: luminosity uncertainty; and
6. $${{\mathrm{cov}}}_\mathrm {PDF}$$: PDF uncertainty.

All JES, unfolding, and luminosity uncertainties are treated as 100 % correlated across the $p_{\mathrm {T}}$ and $|y |$ bins, with the exception of the single-particle response JES source as described in Sect. 2.3. The JES, unfolding, and luminosity uncertainties are treated as multiplicative to avoid the statistical bias that arises when estimating uncertainties from data [45–47].

**Table 2:** Determination of $\alpha _S(M_{\mathrm{Z}})$ in bins of rapidity using the CT10-NLO PDF set. The last row presents the result of a simultaneous fit in all rapidity bins

$$|y | $$ range	No. of data points	$$\alpha _S(M_{\mathrm{Z}})$$	$$\chi ^2/n_\mathrm {dof}$$
$|y | <0.5$	33	$0.1189 \pm 0.0024\,(\text {exp}) \pm 0.0030\,(\mathrm {PDF})$	16.2 / 32
		$$\,\pm \, 0.0008\,(\mathrm {NP}) ^{+0.0045}_{-0.0027}\,(\text {scale})$$	
$0.5\le |y | <1.0$	30	$0.1182 \pm 0.0024\,(\text {exp}) \pm 0.0029\,(\mathrm {PDF})$	25.4 / 29
		$$\,\pm \, 0.0008\,(\mathrm {NP}) ^{+0.0050}_{-0.0025}\,(\text {scale})$$	
$1.0\le |y | <1.5$	27	$0.1165 \pm 0.0027\,(\text {exp}) \pm 0.0024\,(\mathrm {PDF})$	9.5 / 26
		$$\,\pm \, 0.0008\,(\mathrm {NP}) ^{+0.0043}_{-0.0020}\,(\text {scale})$$	
$1.5\le |y | <2.0$	24	$0.1146 \pm 0.0035\,(\text {exp}) \pm 0.0031\,(\mathrm {PDF})$	20.2 / 23
		$$\,\pm \, 0.0013\,(\mathrm {NP}) ^{+0.0037}_{-0.0020}\,(\text {scale})$$	
$2.0\le |y | <2.5$	19	$0.1161 \pm 0.0045\,(\text {exp}) \pm 0.0054\,(\mathrm {PDF})$	12.6 / 18
		$$\,\pm \, 0.0015\,(\mathrm {NP}) ^{+0.0034}_{-0.0032}\,(\text {scale})$$	
$|y | <2.5$	133	$0.1185 \pm 0.0019\,(\text {exp}) \pm 0.0028\,(\mathrm {PDF})$	104.1 / 132
		$$\,\pm \, 0.0004\,(\mathrm {NP}) ^{+0.0053}_{-0.0024}\,(\text {scale})$$	

The derivation of PDF uncertainties follows prescriptions for each individual PDF set. The CT10 and MSTW PDF sets both employ the eigenvector method with upward and downward variations for each eigenvector. As required by the use of covariance matrices, symmetric PDF uncertainties are computed following Ref. [39]. The NNPDF2.1 PDF set uses the MC pseudo-experiments instead of the eigenvector method in order to provide PDF uncertainties. A hundred so-called replicas, whose averaged predictions give the central result, are evaluated following the prescription in Ref. [48] to derive the PDF uncertainty for NNPDF.

As described in Sect. 3.4.1, the NP correction is defined as the centre of the envelope given by pythia6, herwig++, and the powheg$+$pythia6 average of tunes Z2* and P11. Half the spread among these three numbers is taken as the uncertainty. This is the default NP correction used in this analysis. Alternatively, the PS correction factor, defined in Sect. 3.4.2, is applied in addition as an illustrative test to complement the main results.

The uncertainty in $\alpha _S(M_{\mathrm{Z}})$ due to the NP uncertainties is evaluated by looking for maximal offsets from a default fit. The theoretical prediction *T* is varied by the NP uncertainty $\Delta \mathrm {NP}$ as $T\cdot \mathrm {NP} \rightarrow T\cdot \left( \mathrm {NP} \pm \Delta \mathrm {NP}\right)$. The fitting procedure is repeated for these variations, and the deviation from the central $\alpha _S(M_{\mathrm{Z}})$ values is considered as the uncertainty in $\alpha _S(M_{\mathrm{Z}})$.

Finally the uncertainty due to the renormalization and factorisation scales is evaluated by applying the same method as for the NP corrections: $\mu _r$ and $\mu _f$ are varied from the default choice of $\mu _r =\mu _f =p_{\mathrm {T}}$ between $p_{\mathrm {T}}/2$ and $2p_{\mathrm {T}}$ in the following six combinations: $(\mu _r/p_{\mathrm {T}},\mu _f/p_{\mathrm {T}}) = (1/2,1/2)$, (1 / 2, 1), (1, 1 / 2), (1, 2), (2, 1), and (2, 2). The $\chi ^2$ minimisation with respect to $\alpha _S(M_{\mathrm{Z}})$ is repeated in each case. The contribution from the $\mu _r$ and $\mu _f$ scale variations to the uncertainty is evaluated by considering the maximal upwards and downwards deviation of $\alpha _S(M_{\mathrm{Z}})$ from the central result.

### The results on $\alpha _S(M_{\mathrm{Z}})$

**Fig. 9:**
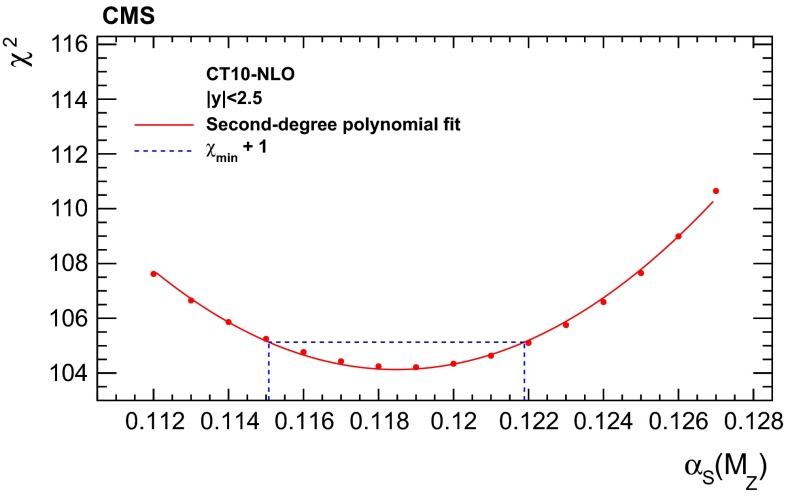
The $\chi ^2$ minimisation with respect to $\alpha _S(M_{\mathrm{Z}})$ using the CT10-NLO PDF set and data from all rapidity bins. The experimental uncertainty is obtained from the $\alpha _S(M_{\mathrm{Z}})$ values for which $\chi ^2$ is increased by one with respect to the minimum value, indicated by the *dashed line*. The *curve* corresponds to a second-degree polynomial fit through the available $\chi ^2$ points

The values of $\alpha _S(M_{\mathrm{Z}})$ obtained with the CT10-NLO PDF set are listed in Table 2 together with the experimental, PDF, NP, and scale uncertainties for each bin in rapidity and for a simultaneous fit of all rapidity bins. To disentangle the uncertainties of experimental origin from those of the PDFs, additional fits without the latter uncertainty source are performed. An example for the evaluation of the uncertainties in a $\chi ^{2}$ fit is shown in Fig. 9. The NP and scale uncertainties are determined via separate fits, as explained above.

For the two outer rapidity bins ($1.5<|y | <2.0$ and $2.0<|y | <2.5$) the series in values of $\alpha _S(M_{\mathrm{Z}})$ of the CT10-NLO PDF set does not reach to sufficiently low values of $\alpha _S(M_{\mathrm{Z}})$. As a consequence the shape of the $\chi ^2$ curve at minimum up to $\chi ^2 +1$ can not be determined completely. To avoid extrapolations based on a polynomial fit to the available points, the alternative $\alpha _S$ evolution code of the HOPPET package [49] is employed. This is the same evolution code as chosen for the creation of the CT10 PDF set. Replacing the original $\alpha _S$ evolution in CT10 by HOPPET, $\alpha _S(M_{\mathrm{Z}})$ can be set freely and in particular different from the default value used in a PDF set, but at the expense of losing the correlation between the value of $\alpha _S(M_{\mathrm{Z}})$ and the fitted PDFs. Downwards or upwards deviations from the lowest and highest values of $\alpha _S(M_{\mathrm{Z}})$, respectively, provided in a PDF series are accepted for uncertainty evaluations up to a limit of $|\Delta \alpha _S(M_{\mathrm{Z}}) | = 0.003$. Applying this method for comparisons, within the available range of $\alpha _S(M_{\mathrm{Z}})$ values, an additional uncertainty is estimated to be negligible.

For comparison the CT10-NNLO PDF set is used for the determination of $\alpha _S(M_{\mathrm{Z}})$. These results are presented in Table 3.

**Table 3:** Determination of $\alpha _S(M_{\mathrm{Z}})$ in bins of rapidity using the CT10-NNLO PDF set. The last row presents the result of a simultaneous fit in all rapidity bins

$$|y | $$ range	No. of data points	$$\alpha _S(M_{\mathrm{Z}})$$	$$\chi ^2/n_\mathrm {dof}$$
$|y | <0.5$	33	$0.1180 \pm 0.0017\,(\text {exp}) \pm 0.0027\,(\mathrm {PDF})$	15.4 / 32
		$$\,\pm \, 0.0006\,(\mathrm {NP})^{+0.0031}_{-0.0026}\,(\text {scale})$$	
$0.5\le |y | <1.0$	30	$0.1176 \pm 0.0016\,(\text {exp}) \pm 0.0026\,(\mathrm {PDF})$	23.9 / 29
		$$\,\pm \, 0.0006\,(\mathrm {NP}) ^{+0.0033}_{-0.0023}\,(\text {scale})$$	
$1.0\le |y | <1.5$	27	$0.1169 \pm 0.0019\,(\text {exp}) \pm 0.0024\,(\mathrm {PDF})$	10.5 / 26
		$$\,\pm \, 0.0006\,(\mathrm {NP}) ^{+0.0033}_{-0.0019}\,(\text {scale})$$	
$1.5\le |y | <2.0$	24	$0.1133 \pm 0.0023\,(\text {exp}) \pm 0.0028\,(\mathrm {PDF})$	22.3 / 23
		$$\,\pm \, 0.0010\,(\mathrm {NP}) ^{+0.0039}_{-0.0029}\,(\text {scale})$$	
$2.0\le |y | <2.5$	19	$0.1172 \pm 0.0044\,(\text {exp}) \pm 0.0039\,(\mathrm {PDF})$	13.8 / 18
		$$\,\pm \, 0.0015\,(\mathrm {NP}) ^{+0.0049}_{-0.0060}\,(\text {scale})$$	
$|y | <2.5$	133	$0.1170 \pm 0.0012\,(\text {exp}) \pm 0.0024\,(\mathrm {PDF})$	105.7 / 132
		$$\,\pm \, 0.0004\,(\mathrm {NP}) ^{+0.0044}_{-0.0030}\,(\text {scale})$$

**Table 4:** Determination of $\alpha _S(M_{\mathrm{Z}})$ using the CT10 and MSTW2008 PDF sets at NLO and the CT10, NNPDF2.1, MSTW2008 PDF sets at NNLO. The results are obtained by a simultaneous fit to all rapidity bins

PDF set	$\alpha _S(M_{\mathrm{Z}})$	$\chi ^2/n_\mathrm {dof}$
CT10-NLO	$0.1185 \pm 0.0019\,(\text {exp}) \pm 0.0028\,(\mathrm {PDF})$ $\,\pm \,0.0004\,(\mathrm {NP})^{+0.0053}_{-0.0024}\,(\text {scale})$	104.1/132
NNPDF2.1-NLO	$0.1150 \pm 0.0015\,(\text {exp}) \pm 0.0024\,(\mathrm {PDF})$ $\,\pm \,0.0003\,(\mathrm {NP})^{+0.0025}_{-0.0025}\,(\text {scale})$	103.5/132
MSTW2008-NLO	$0.1159 \pm 0.0012\,(\text {exp}) \pm 0.0014\,(\mathrm {PDF})$ $\,\pm \,0.0001\,(\mathrm {NP})^{+0.0024}_{-0.0030}\,(\text {scale})$	107.9/132
CT10-NNLO	$0.1170 \pm 0.0012\,(\text {exp}) \pm 0.0024\,(\mathrm {PDF})$ $\,\pm \,0.0004\,(\mathrm {NP})^{+0.0044}_{-0.0030}\,(\text {scale})$	105.7/132
NNPDF2.1-NNLO	$0.1175 \pm 0.0012\,(\text {exp}) \pm 0.0019\,(\mathrm {PDF})$ $\,\pm \,0.0001\,(\mathrm {NP})^{+0.0018}_{-0.0020}\,(\text {scale})$	103.0/132
MSTW2008-NNLO	$0.1136 \pm 0.0010\,(\text {exp}) \pm 0.0011\,(\mathrm {PDF})$ $\,\pm \,0.0001\,(\mathrm {NP})^{+0.0019}_{-0.0024}\,(\text {scale})$	108.8 / 132

The final result using all rapidity bins and the CT10-NLO PDF set is (last row of Table 2)

11 $$\begin{aligned} \alpha _S(M_{\mathrm{Z}})= & {} 0.1185 \pm 0.0019\,\text {(exp)} \nonumber \\&\pm 0.0028\,(\mathrm {PDF}) \pm 0.0004\,(\mathrm {NP})^{+0.0053}_{-0.0024}\,(\text {scale}) \nonumber \\= & {} 0.1185 \pm 0.0034\,\text {(all except scale)}^{+0.0053}_{-0.0024}\,(\text {scale}) \nonumber \\= & {} 0.1185^{+0.0063}_{-0.0042}, \end{aligned}$$

where experimental, PDF, NP, and scale uncertainties have been added quadratically to give the total uncertainty. The result is in agreement with the world average value of $\alpha _S(M_{\mathrm{Z}}) = 0.1185 \pm 0.0006$ [50], with the Tevatron results [51–53], and recent results obtained with LHC data [54–56]. The determination of $\alpha _S(M_{\mathrm{Z}})$, which is based on the CT10-NLO PDF set, is also in agreement with the result obtained using the NNPDF2.1-NLO and MSTW2008-NLO sets, as shown in Table 4. For comparison this table also shows the results using the CT10, MSTW2008, and NNPDF2.1 PDF sets at NNLO. The $\alpha _S(M_{\mathrm{Z}})$ values are in agreement among the different NLO PDF sets within the uncertainties.

Applying the PS correction factor to the NLO theory prediction in addition to the NP correction as discussed in Sect. 3.4.2, the fit using all rapidity bins and the CT10-NLO PDF set yields $\alpha _S(M_{\mathrm{Z}}) = 0.1204 \pm 0.0018\,(\text {exp})$. This value is in agreement with our main result of Eq. (11), which is obtained using only the NP correction factor.

To investigate the running of the strong coupling, the fitted region is split into six bins of $p_{\mathrm {T}}$ and the fitting procedure is repeated in each of these bins. The six extractions of $\alpha _S(M_{\mathrm{Z}})$ are reported in Table 5. The $\alpha _S(M_{\mathrm{Z}})$ values are evolved to the corresponding energy scale *Q* using the two-loop solution to the renormalization group equation (RGE) within HOPPET. The value of *Q* is calculated as a cross section weighted average in each fit region. These average scale values *Q*, derived again with the fastNLO framework, are identical within about 1$\,\text {GeV}$ for different PDFs. To emphasise that theoretical uncertainties limit the achievable precision, Tables 6 and 7 present for the six bins in $p_{\mathrm {T}}$ the total uncertainty as well as the experimental, PDF, NP, and scale components, where the six experimental uncertainties are all correlated.

**Table 5:** Determination of $\alpha _S$ in separate bins of jet $p_{\mathrm {T}}$ using the CT10-NLO PDF set

$p_{\mathrm {T}}$ range ($\text {GeV}$ )	*Q* ($\text {GeV}$ )	$\alpha _S(M_{\mathrm{Z}})$	$\alpha _S(Q)$	No. of data points	$\chi ^2/n_\mathrm {dof}$
114–196	136	$0.1172_{-0.0043}^{+0.0058}$	$0.1106^{+0.0052}_{-0.0038}$	20	6.2 / 19
196–300	226	$0.1180_{-0.0046}^{+0.0063}$	$0.1038^{+0.0048}_{-0.0035}$	20	7.6 / 19
300–468	345	$0.1194_{-0.0049}^{+0.0064}$	$0.0993^{+0.0044}_{-0.0034}$	25	8.1 / 24
468–638	521	$0.1187_{-0.0051}^{+0.0067}$	$0.0940^{+0.0041}_{-0.0032}$	20	10.6 / 19
638–905	711	$0.1192_{-0.0056}^{+0.0074}$	$0.0909^{+0.0042}_{-0.0033}$	22	11.2 / 21
905–2116	1007	$0.1176_{-0.0065}^{+0.0111}$	$0.0866^{+0.0057}_{-0.0036}$	26	33.6 / 25

**Table 6:** Uncertainty composition for $\alpha _S(M_{\mathrm{Z}})$ from the determination of $\alpha _S(Q)$ in bins of $p_{\mathrm {T}}$ using the CT10-NLO PDF set

$p_{\mathrm {T}}$ range ($\text {GeV}$)	*Q* ($\text {GeV}$ )	$\alpha _S(M_{\mathrm{Z}})$	Exp.	PDF	NP	Scale
114–196	136	0.1172	$\pm {0.0031}$	$\pm {0.0018}$	$\pm {0.0007}$	$_{-0.0022}^{+0.0045}$
196–300	226	0.1180	$\pm {0.0034}$	$\pm {0.0019}$	$\pm {0.0011}$	$_{-0.0025}^{+0.0048}$
300–468	345	0.1194	$\pm {0.0032}$	$\pm {0.0023}$	$\pm {0.0010}$	$_{-0.0027}^{+0.0049}$
468–638	521	0.1187	$\pm {0.0029}$	$\pm {0.0031}$	$\pm {0.0006}$	$_{-0.0027}^{+0.0052}$
638–905	711	0.1192	$\pm {0.0034}$	$\pm {0.0032}$	$\pm {0.0005}$	$_{-0.0030}^{+0.0057}$
905–2116	1007	0.1176	$\pm {0.0047}$	$\pm {0.0040}$	$\pm {0.0002}$	$_{-0.0020}^{+0.0092}$

**Table 7:** Uncertainty composition for $\alpha _S(Q)$ in bins of $p_{\mathrm {T}}$ using the CT10-NLO PDF set

$p_{\mathrm {T}}$ range ($\text {GeV}$)	*Q* ($\text {GeV}$ )	$\alpha _S(Q)$	Exp.	PDF	NP	Scale
114–196	136	0.1106	$\pm {0.0028}$	$\pm {0.0016}$	$\pm {0.0006}$	$_{-0.0020}^{+0.0040}$
196–300	226	0.1038	$\pm {0.0026}$	$\pm {0.0015}$	$\pm {0.0008}$	$_{-0.0019}^{+0.0037}$
300–468	345	0.0993	$\pm {0.0022}$	$\pm {0.0016}$	$\pm {0.0007}$	$_{-0.0019}^{+0.0033}$
468–638	521	0.0940	$\pm {0.0018}$	$\pm {0.0019}$	$\pm {0.0004}$	$_{-0.0017}^{+0.0032}$
638–905	711	0.0909	$\pm {0.0019}$	$\pm {0.0018}$	$\pm {0.0003}$	$_{-0.0017}^{+0.0032}$
905–2116	1007	0.0866	$\pm {0.0025}$	$\pm {0.0021}$	$\pm {0.0001}$	$_{-0.0011}^{+0.0048}$

**Fig. 10:**
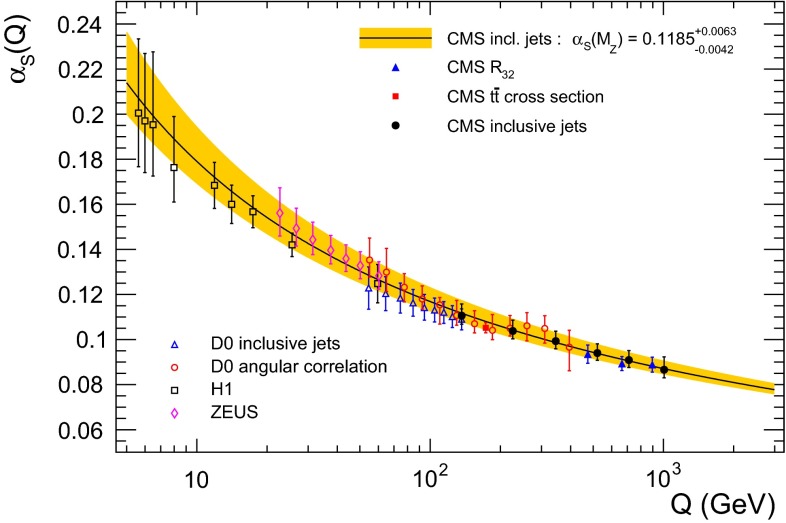
The strong coupling $\alpha _S(Q)$ (*full line*) and its total uncertainty (*band*) as determined in this analysis using a two-loop solution to the RGE as a function of the momentum transfer $Q=p_{\mathrm {T}}$. The extractions of $\alpha _S(Q)$ in six separate ranges of *Q* as presented in Table 5 are shown together with results from the H1 [58, 59], ZEUS [60], and D0 [52, 53] experiments at the HERA and Tevatron colliders. Other recent CMS measurements [55, 56] are displayed as well. The uncertainties represented by *error bars* are subject to correlations

Figure 10 presents the running of the strong coupling $\alpha _S(Q)$ and its total uncertainty as determined in this analysis. The extractions of $\alpha _S(Q)$ in six separate ranges of *Q*, as presented in Table 5, are also shown. In the same figure the values of $\alpha _S$ at lower scales determined by the H1 [57–59], ZEUS [60], and D0 [52, 53] collaborations are shown for comparison. Recent CMS measurements [55, 56], which are in agreement with the $\alpha _S(M_{\mathrm{Z}})$ determination of this study, are displayed as well. The results on $\alpha _S$ reported here are consistent with the energy dependence predicted by the RGE.

## Study of PDF constraints with HERAFitter

The PDFs of the proton are an essential ingredient for precision studies in hadron-induced reactions. They are derived from experimental data involving collider and fixed-target experiments. The DIS data from the HERA-I $$\mathrm {e}$$$$\mathrm {p}$$ collider cover most of the kinematic phase space needed for a reliable PDF extraction. The $$\mathrm {p}$$$$\mathrm {p}$$ inclusive jet cross section contains additional information that can constrain the PDFs, in particular the gluon, in the region of high fractions *x* of the proton momentum.

The HERAFitter project [61, 62] is an open-source framework designed among other things to fit PDFs to data. It has a modular structure, encompassing a variety of theoretical predictions for different processes and phenomenological approaches for determining the parameters of the PDFs. In this study, the recently updated HERAFitter version 1.1.1 is employed to estimate the impact of the CMS inclusive jet data on the PDFs and their uncertainties. Theory is used at NLO for both processes, i.e. up to order $$\alpha _S ^2$$ for DIS and up to order $$\alpha _S ^3$$ for inclusive jet production in $$\mathrm {p}$$$$\mathrm {p}$$ collisions.

### Correlation between inclusive jet production and the PDFs

The potential impact of the CMS inclusive jet data can be illustrated by the correlation between the inclusive jet cross section $\sigma _{\text {jet}}(Q)$ and the PDF $xf(x,Q^2)$ for any parton flavour *f*. The NNPDF Collaboration [63] provides PDF sets in the form of an ensemble of replicas *i*, which sample variations in the PDF parameter space within allowed uncertainties. The correlation coefficient $\varrho _f(x,Q)$ between a cross section and the PDF for flavour *f* at a point (*x*, *Q*) can be computed by evaluating means and standard deviations from an ensemble of *N* replicas as

12 $$\begin{aligned}&\varrho _f (x,Q) = \frac{N}{(N-1)}\nonumber \\&\quad \frac{ \langle \sigma _{\text {jet}}(Q)_i \cdot xf(x,Q^2)_i \rangle - \langle \sigma _{\text {jet}}(Q)_i \rangle \cdot \langle xf(x,Q^2)_i \rangle }{\Delta _{\sigma _{\text {jet}}(Q)} \Delta _{xf(x,Q^2)}}.\qquad \end{aligned}$$

**Fig. 11:**
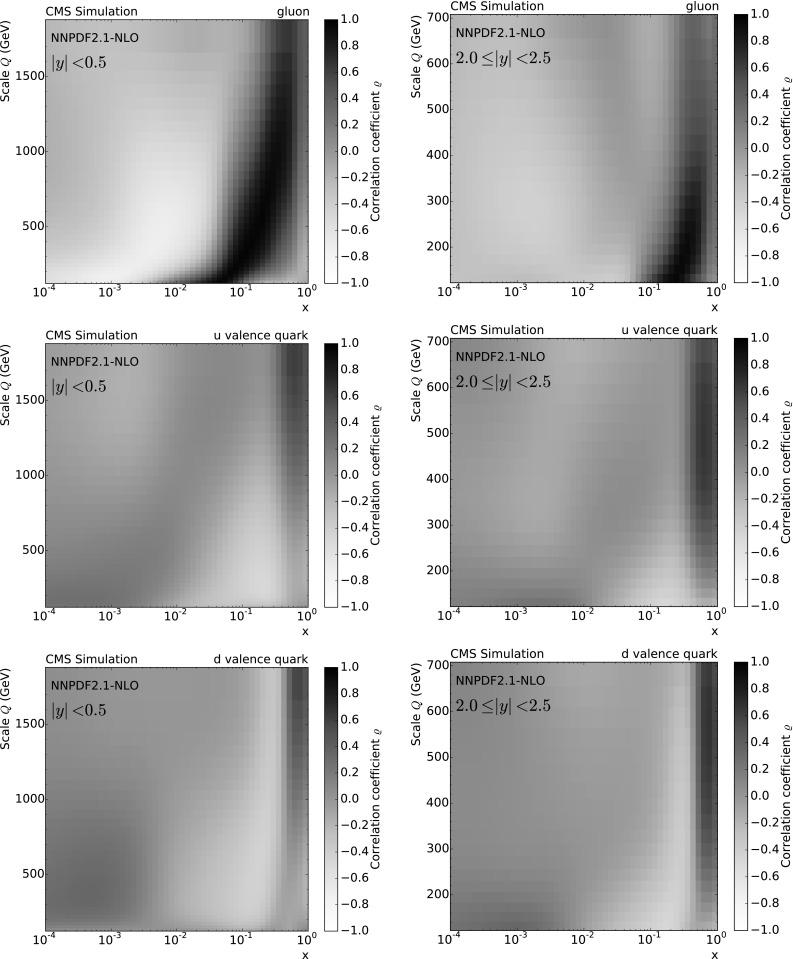
The correlation coefficient between the inclusive jet cross section and the gluon (*top row*), the u valence quark (*middle row*), and the d valence quark PDFs (*bottom row*), as a function of the momentum fraction *x* of the proton and the energy scale *Q* of the hard process. The correlation is shown for the central rapidity region $|y | <0.5$ (*left*) and for $2.0<|y | <2.5$ (*right*)

Here, the angular brackets denote the averaging over the replica index *i*, and $\Delta$ represents the evaluation of the corresponding standard deviation for either the jet cross section, $\Delta _{\sigma _{\text {jet}}(Q)}$, or a PDF, $\Delta _{xf(x,Q^2)}$. Figure 11 presents the correlation coefficient between the inclusive jet cross section and the gluon, u valence quark, and d valence quark PDFs in the proton.

The correlation between the gluon PDF and the inclusive jet cross section is largest at central rapidity for most jet $p_{\mathrm {T}}$. In contrast, the correlation between the valence quark distributions and the jet cross section is rather small except for very high $p_{\mathrm {T}}$ such that some impact can be expected at high *x* from including these jet data in PDF fits. In the forward region the correlation between the valence quark distributions and the jet cross sections is more pronounced at high *x* and smaller jet $p_{\mathrm {T}}$. Therefore, a significant reduction of the PDF uncertainties is expected by including the CMS inclusive jet cross section into fits of the proton structure.

### The fitting framework

#### The HERAFitter setup

The impact of the CMS inclusive jet data on proton PDFs is investigated by including the jet cross section measurement in a combined fit at NLO with the HERA-I inclusive DIS cross sections [19], which were the basis for the determination of the HERAPDF1.0 PDF set. The analysis is performed within the HERAFitter framework using the Dokshitzer–Gribov–Lipatov–Altarelli–Parisi [64–66] evolution scheme at NLO as implemented in the QCDNUM package [67] and the generalised-mass variable-flavour number Thorne–Roberts scheme [68, 69].

In contrast to the original HERAPDF fit, the results presented here require the DIS data to fulfill $Q^2 > Q_\text {min}^2 = 7.5 \,\text {GeV} ^2$ instead of $3.5\,\text {GeV} ^2$. The amount of DIS data left out by the increased $Q_\text {min}^2$ threshold is rather small and concerns a phase space where a perturbative description is less reliable. A similar, higher cutoff has been applied by the ATLAS Collaboration [70, 71]. As a crosscheck all fits have been performed for a cutoff of $Q^2 > Q_\text {min}^2 = 3.5 \,\text {GeV} ^2$, and the results are consistent with the ones obtained using the more stringent cutoff. Differences beyond the expected reduction of uncertainties at low *x* have not been observed.

The following PDFs are independent in the fit procedure: $xu_v(x)$, $xd_v(x)$, *xg*(*x*), and $x\overline{U}(x)$, $x\overline{D}(x)$, where $x\overline{U}(x) = x\overline{u}(x)$, and $x\overline{D}(x) = x\overline{d}(x) + x\overline{s}(x)$. Similar to Ref. [72], a parameterization with 13 free parameters is used. At the starting scale $Q_0$ of the QCD evolution, chosen to be $Q_0^2 = 1.9 \,\text {GeV} ^2$, the PDFs are parameterized as follows:

13 $$\begin{aligned} xg(x)&= A_g x^{B_g} (1-x)^{C_g} - A'_g x^{B'_g} (1-x)^{C'_g}, \nonumber \\ xu_v(x)&= A_{u_{v}} x^{B_{u_{v}}} (1-x)^{C_{u_{v}}} (1 + E_{u_{v}}x^2), \nonumber \\ xd_v(x)&= A_{d_v} x^{B_{d_v}} (1-x)^{C_{d_{v}}}, \\ x\overline{U}(x)&= A_{\overline{U}} x^{B_{\overline{U}}} (1-x)^{C_{\overline{U}}}, \text {and} \nonumber \\ x\overline{D}(x)&= A_{\overline{D}} x^{B_{\overline{D}}} (1-x)^{C_{\overline{D}}}. \nonumber \end{aligned}$$

The normalisation parameters $A_g$, $A_{u_{v}}$, and $A_{d_{v}}$ are constrained by QCD sum rules. Additional constraints $B_{\overline{U}}=B_{\overline{D}}$ and $A_{\overline{U}} = A_{\overline{D}}(1-f_s)$ are applied to ensure the same normalisation for the $\overline{u}$ and $\overline{d}$ densities for $x \rightarrow 0$. The strangeness fraction is set to $f_s = 0.31$, as obtained from neutrino-induced dimuon production [73]. The parameter $C'_g$ is fixed to 25 [20, 69] and the strong coupling constant to $\alpha _S(M_{\mathrm{Z}}) = 0.1176$.

**Table 8:** The 19 independent sources of systematic uncertainty considered in the CMS inclusive jet measurement. Out of these, 16 are related to the JES and are listed first. In order to implement the improved correlation treatment as described in Sect. 2.3, the single-particle response source JEC2, see also Appendix A, has been split up into five sources: JEC2a–JEC2e. The shift from the default value in each source of systematic uncertainty is determined by nuisance parameters in the fit and is presented in units of standard deviations

Systematic source	Shift in standard deviations
JEC0 absolute jet energy scale	0.09
JEC1 MC extrapolation	0.00
JEC2a single-particle response barrel	1.31
JEC2b single-particle response endcap	$-1.46$
JEC2c single-particle decorrelation $|y | <0.5$	0.20
JEC2d single-particle decorrelation $0.5\le |y | <1.0$	0.19
JEC2e single-particle decorrelation $1.0\le |y | <1.5$	0.92
JEC3 jet flavor correction	0.04
JEC4 time-dependent detector effects	$-0.15$
JEC5 jet $p_{\mathrm {T}}$ resolution in endcap 1	0.76
JEC6 jet $p_{\mathrm {T}}$ resolution in endcap 2	$-0.42$
JEC7 jet $p_{\mathrm {T}}$ resolution in HF	0.01
JEC8 correction for final-state radiation	0.03
JEC9 statistical uncertainty of $\eta$-dependent correction for endcap	$-0.42$
JEC10 statistical uncertainty of $\eta$-dependent correction for HF	0.00
JEC11 data-MC difference in $\eta$-dependent pileup correction	0.91
JEC12 residual out-of-time pileup correction for prescaled triggers	$-0.17$
JEC13 offset dependence in pileup correction	$-0.03$
JEC14 MC pileup bias correction	0.39
JEC15 jet rate dependent pileup correction	0.29
Unfolding	$-0.26$
Luminosity	$-0.07$
NP correction	0.60

#### Definition of the goodness-of-fit estimator

The agreement between the *N* data points $D_i$ and the theoretical predictions $T_i$ is quantified via a least-squares method, where

14 $$\begin{aligned} \chi ^2&= \sum _{ij}^N \left( D_i - T_i - \sum _k^K r_k \beta _{ik}\right) \nonumber \\&\quad \times \mathrm {C}_{ij}^{-1} \left( D_j - T_j - \sum _k^K r_k \beta _{jk} \right) + \sum _k^K r_k^2. \end{aligned}$$

For fully correlated sources of uncertainty following a Gaussian distribution with a zero mean and a root-mean-square of unity as assumed here, this definition is equivalent to Eq. (9) [74]. As a bonus, the systematic shift of the nuisance parameter $r_k$ for each source in a fit is determined. Numerous large shifts in either direction indicate a problem as for example observed while fitting $\alpha _S(M_{\mathrm{Z}})$ with this technique and the old uncertainty correlation prescription.

In the following, the covariance matrix is defined as $\mathrm {C} = {{\mathrm{cov}}}_{\text {stat}} + {{\mathrm{cov}}}_{\text {uncor}}$, while the JES, unfolding, and luminosity determination are treated as fully correlated systematic uncertainties $\beta _{ik}$ with nuisance parameters $r_k$. Including also the NP uncertainties, treated via the offset method in Sect. 4, in the form of one nuisance parameter in total *K* such sources are defined. Of course, PDF uncertainties emerge as results of the fits performed here, in contrast to serving as inputs, as they do in the fits of $\alpha _S(M_{\mathrm{Z}})$ presented in Sect. 4.

**Table 9:** Partial $\chi ^2$ values, $\chi ^2_\mathrm {p}$, for each data set in the HERA-I DIS (middle section) or in the combined fit including CMS inclusive jet data (right section). Here, $n_{\mathrm {data}}$ is the number of data points available for the determination of the 13 parameters. The bottom two lines show the total $\chi ^2$ and $\chi ^2/n_\mathrm {dof}$. The difference between the sum of all $\chi ^2_\mathrm {p}$ and the total $\chi ^2$ for the combined fit is attributed to the nuisance parameters

Data set	$n_{\mathrm {data}}$	HERA-I data		HERA-I and CMS data	
		$\chi ^2_\mathrm {p}$	$\chi ^2_\mathrm {p}/n_\mathrm {data}$	$\chi ^2_\mathrm {p}$	$\chi ^2_\mathrm {p}/n_\mathrm {data}$
NC HERA-I H1-ZEUS combined $\mathrm {e}^-\mathrm {p}$	145	109	0.75	109	0.75
NC HERA-I H1-ZEUS combined $\mathrm {e}^+\mathrm {p}$	337	309	0.91	311	0.92
CC HERA-I H1-ZEUS combined $\mathrm {e}^-\mathrm {p}$	34	20	0.59	22	0.65
CC HERA-I H1-ZEUS combined $\mathrm {e}^+\mathrm {p}$	34	29	0.85	35	1.03
CMS inclusive jets	133	–	–	102	0.77

Data set(s)	$n_{\mathrm {dof}}$	$\chi ^2$	$\chi ^2/n_\mathrm {dof}$	$\chi ^2$	$\chi ^2/n_\mathrm {dof}$
HERA-I data	537	468	0.87	–	–
HERA-I and CMS data	670	–	–	591	0.88

All the fully correlated sources are assumed to be multiplicative to avoid the statistical bias that arises from uncertainty estimations taken from data [45–47]. As a consequence, the covariance matrix of the remaining sources has to be re-evaluated in each iteration step. To inhibit the compensation of large systematic shifts by increasing simultaneously the theoretical prediction and the statistical uncertainties, the systematic shifts of the theory are taken into account before the rescaling of the statistical uncertainty. Otherwise alternative minima in $\chi ^2$ can appear that are associated with large theoretical predictions and correspondingly large shifts in the nuisance parameters. These alternative minima are clearly undesirable [62].

**Fig. 12:**
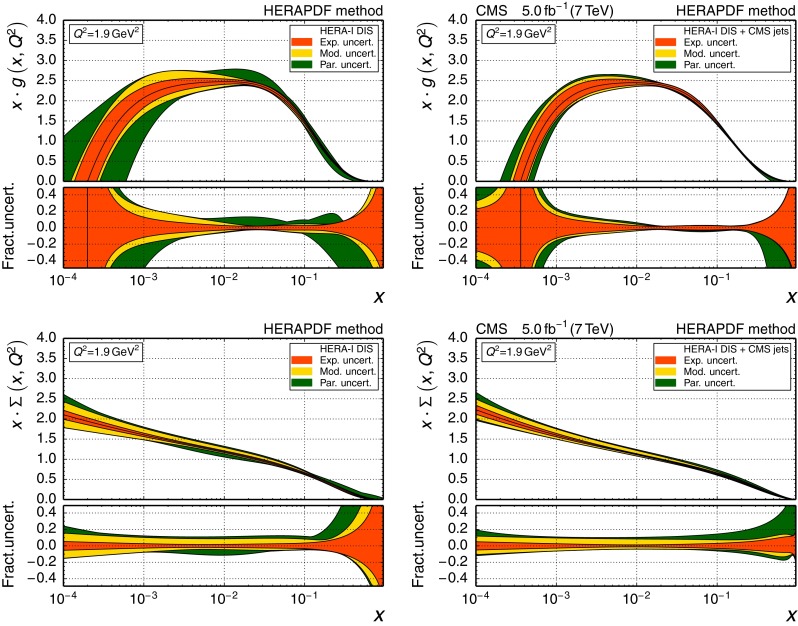
The gluon (*top*) and sea quark (*bottom*) PDFs as a function of *x* as derived from HERA-I inclusive DIS data alone (*left*) and in combination with CMS inclusive jet data (*right*). The PDFs are shown at the starting scale $Q^2 = 1.9 \,\text {GeV} ^2$. The experimental (*inner band*), model (*middle band*), and parameterization uncertainties (*outer band*) are successively added quadratically to give the total uncertainty

#### Treatment of CMS data uncertainties

The JES is the dominant source of experimental systematic uncertainty in jet cross sections. As described in Sect. 2.3, the $p_{\mathrm {T}}$- and $\eta$-dependent JES uncertainties are split into 16 uncorrelated sources that are fully correlated in $p_{\mathrm {T}}$ and $\eta$. Following the modified recommendation for the correlations versus rapidity of the single-particle response source as given in Sect. 2.3, it is necessary to split this source into five parts for the purpose of using the uncertainties published in Ref. [1] within the $\chi ^2$ fits. The complete set of uncertainty sources is shown in Table 8.

By employing the technique of nuisance parameters, the impact of each systematic source of uncertainty on the fit result can be examined separately. For an adequate estimation of the size and the correlations of all uncertainties, the majority of all systematic sources should be shifted by less than one standard deviation from the default in the fitting procedure. Table 8 demonstrates that this is the case for the CMS inclusive jet data.

**Fig. 13:**
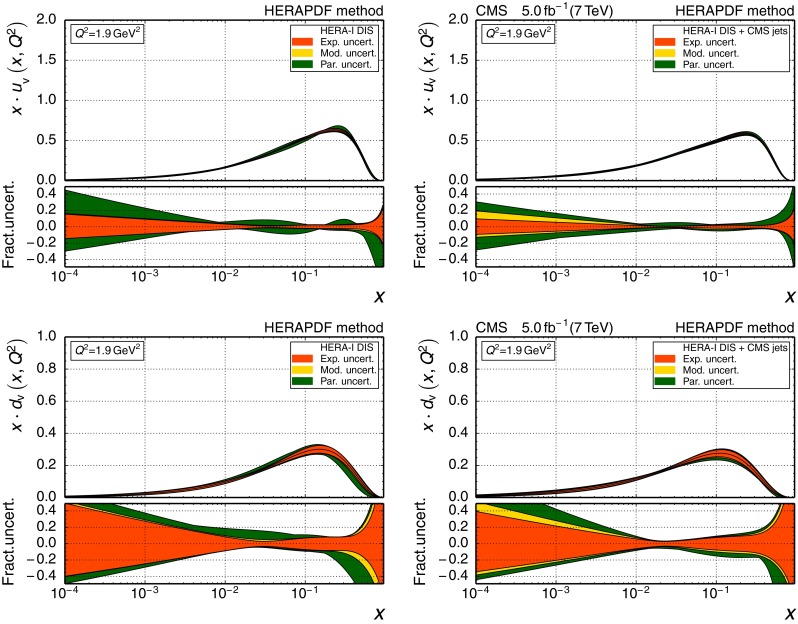
The u valence quark (*top*) and d valence quark (*bottom*) PDFs as a function of *x* as derived from HERA-I inclusive DIS data alone (*left*) and in combination with CMS inclusive jet data (*right*). The PDFs are shown at the starting scale $Q^2 = 1.9 \,\text {GeV} ^2$. The experimental (*inner band*), model (*middle band*), and parameterization uncertainties (*outer band*) are successively added quadratically to give the total uncertainty

In contrast, with the original assumption of full correlation within the 16 JES systematic sources across all $|y |$ bins, shifts beyond two standard deviations were apparent and led to a re-examination of this issue and the improved correlation treatment of the JES uncertainties as described previously in Sect. 2.3.

### Determination of PDF uncertainties according to the HERAPDF prescription

The uncertainty in the PDFs is subdivided into experimental, model, and parameterization uncertainties that are studied separately. In the default setup of the HERAFitter framework, experimental uncertainties are evaluated following a Hessian method [74], and result from the propagated statistical and systematic uncertainties of the input data.

**Fig. 14:**
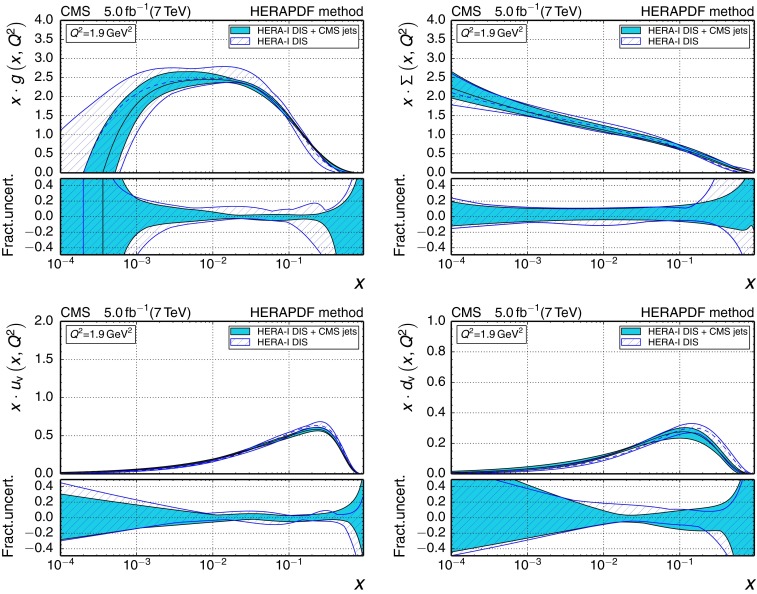
The gluon (*top left*), sea quark (*top right*), u valence quark (*bottom left*), and d valence quark (*bottom right*) PDFs as a function of *x* as derived from HERA-I inclusive DIS data alone (*dashed line*) and in combination with CMS inclusive jet data (*full line*). The PDFs are determined employing the HERAPDF method with a $Q^2_\mathrm {min} = 7.5\,\text {GeV} ^2$ selection criterion. The PDFs are shown at the starting scale $Q^2 = 1.9\,\text {GeV} ^2$. Only the total uncertainty in the PDFs is shown (*hatched* and *solid bands*)

For the model uncertainties, the offset method [75] is applied considering the following variations of model assumptions:

1. The strangeness fraction $f_s$, by default equal to 0.31, is varied between 0.23 and 0.38.
2. The b-quark mass is varied by $\pm 0.25\,\text {GeV}$ around the central value of $4.75\,\text {GeV}$.
3. The c-quark mass, with the central value of $1.4\,\text {GeV}$, is varied to 1.35 and $1.65\,\text {GeV}$. For the downwards variation the charm production threshold is avoided by changing the starting scale to $Q_0^2=1.8\,\text {GeV} ^2$ in this case.
4. The minimum $Q^2$ value for data used in the fit, $Q^2_\mathrm {min}$, is varied from 7.5 to 5.0 and $10\,\text {GeV} ^2$.

The PDF parameterization uncertainty is estimated as described in Ref. [19]. By employing the more general form of parameterizations

15 $$\begin{aligned} xg(x)&= A_g x^{B_g} (1-x)^{C_g} (1 + D_g x + E_g x^2) \nonumber \\&\quad - A'_g x^{B'_g} (1-x)^{C'_g},\\ xf(x)&= A_{f} x^{B_{f}} (1-x)^{C_{f}} (1 + D_{f}x + E_{f}x^2) \nonumber \end{aligned}$$

for gluons and the nongluon flavours, respectively, it is tested whether the successive inclusion of additional fit parameters leads to a variation in the shape of the fitted results. Furthermore, the starting scale $Q_0$ is changed to $Q^2_0 = 1.5$ and $2.5\,\text {GeV} ^2$. The maximal deviations of the resulting PDFs from those obtained in the central fit define the parameterization uncertainty. The experimental, model, and parameterization uncertainties are added in quadrature to give the final PDF uncertainty according to the HERAPDF prescription [19].

Using this fitting setup, the partial $\chi ^2$ values per number of data points, $n_{\mathrm {data}}$, are reported in Table 9 for each of the neutral current (NC) and charged current (CC) data sets in the HERA-I DIS fit and for the combined fit including the CMS inclusive jet data. The achieved fit qualities demonstrate the compatibility of all data within the presented PDF fitting framework. The resulting PDFs with breakdown of the uncertainties for the gluon, the sea, u valence, and d valence quarks with and without CMS inclusive jet data are arranged next to each other in Figs. 12 and 13. Figure 14 provides direct comparisons of the two fit results with total uncertainties. The parameterization and model uncertainties of the gluon distribution are significantly reduced for almost the whole *x* range from $10^{-4}$ up to 0.5. When DIS data below $Q^2_\mathrm {min} = 7.5 \,\text {GeV} ^2$ are included in the fit, the effect is much reduced for the low *x* region $x < 0.01$, but remains important for medium to high *x*. Also, for the u valence, d valence, and sea quark distributions some reduction in their uncertainty is visible at high *x* ($x \gtrsim 0.1$).

At the same time, some structure can be seen, particularly in the parameterization uncertainties that might point to a still insufficient flexibility in the parameterizations. Therefore, a comparison is presented in the next Sect. 5.4, using the MC method with the regularisation based on data, which is also implemented within the HERAFitter framework.

**Fig. 15:**
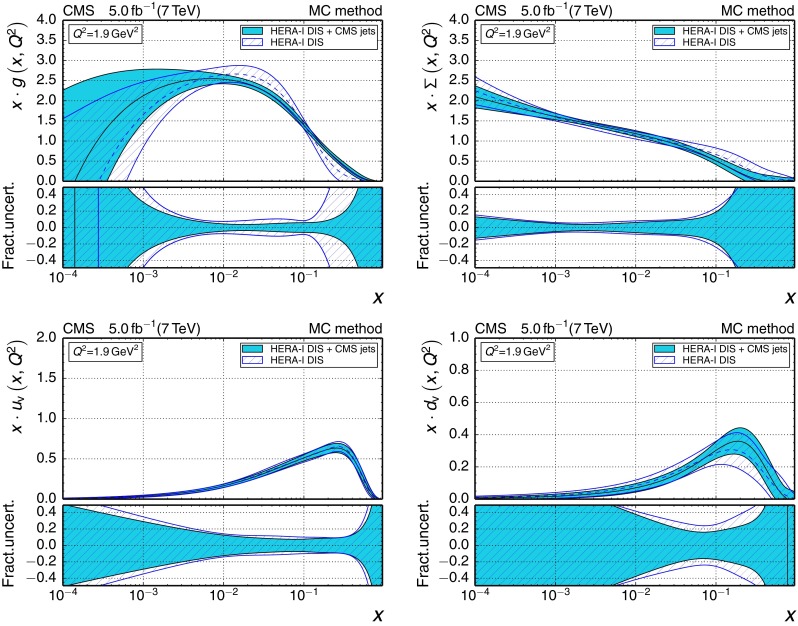
The gluon (*top left*), sea quark (*top right*), u valence quark (*bottom left*), and d valence quark (*bottom right*) PDFs as a function of *x* as derived from HERA-I inclusive DIS data alone (*dashed line*) and in combination with CMS inclusive jet data (*full line*). The PDFs are determined employing the MC method with data-derived regularisation. The PDFs are shown at the starting scale $Q^2 = 1.9\,\text {GeV} ^2$. Only the total uncertainty in the PDFs is shown (*hatched* and *solid bands*)

**Fig. 16:**
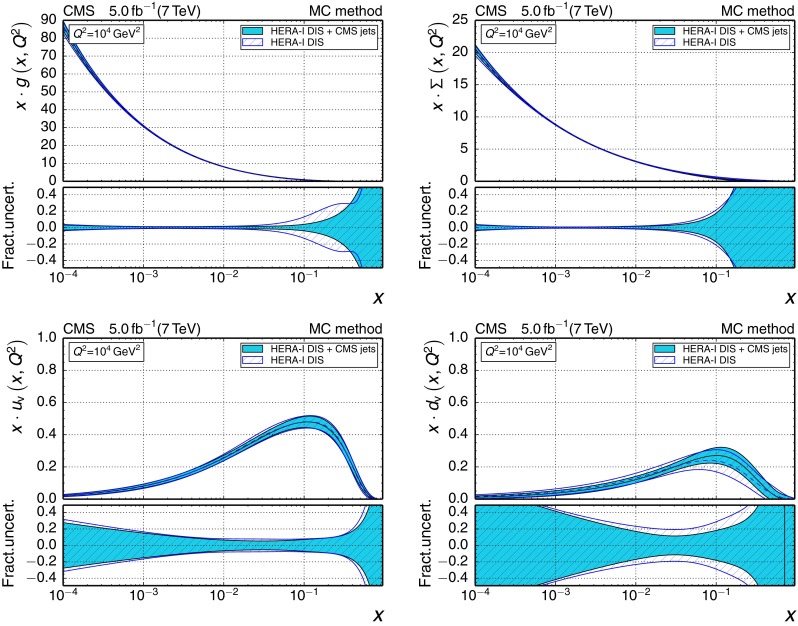
The gluon (*top left*), sea quark (*top right*), u valence quark (*bottom left*), and d valence quark (*bottom right*) PDFs as a function of *x* as derived from HERA-I inclusive DIS data alone (*dashed line*) and in combination with CMS inclusive jet data (*full line*). The PDFs are determined employing the MC method with data-derived regularisation. The PDFs are evolved to $Q^2 = 10^4 \,\text {GeV} ^2$. Only the total uncertainty in the PDFs is shown (*hatched* and *solid bands*)

**Fig. 17:**
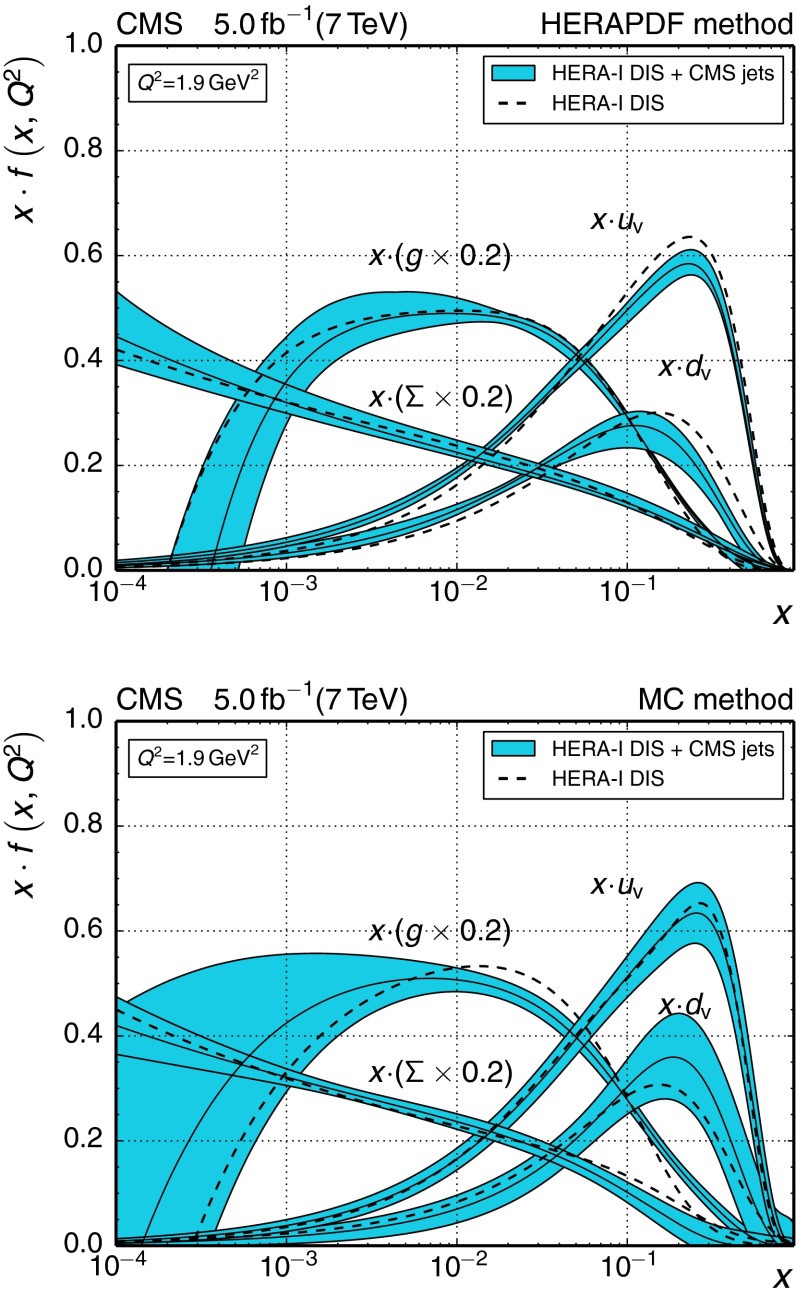
Overview of the gluon, sea, u valence, and d valence PDFs before (*dashed line*) and after (*full line*) including the CMS inclusive jet data into the fit. The *plots* show the PDF fit outcome from the HERAPDF method (*top*) and from the MC method with data-derived regularisation (*bottom*). The PDFs are shown at the starting scale $Q^2 = 1.9 \,\text {GeV} ^2$. The total uncertainty including the CMS inclusive jet data is shown as a band around the central fit result

### Determination of PDF uncertainties using the MC method with regularisation

To study more flexible PDF parameterizations, a MC method based on varying the input data within their correlated uncertainties is employed in combination with a data-based regularisation technique. This method was first used by the NNPDF Collaboration and uses a more flexible parameterization to describe the *x* dependence of the PDFs [63]. To avoid the fitting of statistical fluctuations present in the input data (over-fitting) a data-based stopping criterion is introduced. The data set is split randomly into a “fit” and a “control” sample. The $\chi ^2$ minimisation is performed with the “fit” sample while simultaneously the $\chi ^2$ of the “control” sample is calculated using the current PDF parameters. It is observed that the $\chi ^2$ of the “control” sample at first decreases and then starts to increase again because of over-fitting. At this point, the fit is stopped. This regularisation technique is used in combination with a MC method to estimate the central value and the uncertainties of the fitted PDFs. Before a fit, several hundred replica sets are created by allowing the central values of the measured cross section to fluctuate within their statistical and systematic uncertainties while taking into account all correlations. For each replica, a fit to NLO QCD is performed, which yields an optimum value and uncertainty for each parameter. The collection of all replica fits can then provide an ensemble average and root-mean-square. Moreover, the variations to derive the model dependence of the HERAPDF prescription do not lead to any further increase of the uncertainty.

Similarly to Fig. 14 for the HERAPDF method, a direct comparison of the two fit results with total uncertainties is shown in Fig. 15 for the MC method. The total uncertainty derived with the MC method is almost always larger than with the HERAPDF technique, and in the case of the gluon at low *x*, it is much larger. In both cases a significant reduction of the uncertainty in the gluon PDF is observed, notably in the *x* range from $$10^{-2}$$ up to 0.5. Both methods also lead to a decrease in the gluon PDF between $$10^{-2}$$ and $$10^{-1}$$ and an increase for larger *x*. Although this change is more pronounced when applying the MC method, within the respective uncertainties both results are compatible. For the sea quark only small differences in shape are observed, but, in contrast to the HERAPDF method that exhibits reduced uncertainties for $$x > 0.2$$, this is not visible when using the MC method. Both methods agree on a very modest reduction in uncertainty at high $$x > 0.05$$ in the u valence quark PDF and a somehwat larger improvement for the d valence quark PDF, which is expected from the correlations, studied in Fig. 11, where the quark distributions are constrained via the $${\mathrm{q}}$$$${\mathrm{q}}$$ contribution to jet production at high $$|y |$$ and $$p_{\mathrm {T}}$$. Changes in shape of the d valence quark PDF go into opposite directions for the two methods, but are compatible within uncertainties.

All preceding figures presented the PDFs at the starting scale of the evolution of $Q^2 = 1.9 \,\text {GeV} ^2$. For illustration, Fig. 16 displays the PDFs derived with the regularised MC method after evolution to a scale of $Q^2 = 10^4 \,\text {GeV} ^2$. Finally, Fig. 17 shows an overview of the gluon, sea, u valence, and d valence distributions at the starting scale of $Q^2 = 1.9 \,\text {GeV} ^2$ for both techniques, the HERAPDF and the regularised MC method.

### Combined fit of PDFs and the strong coupling constant

Inclusive DIS data alone are not sufficient to disentangle effects on cross section predictions from changes in the gluon distribution or $\alpha _S(M_{\mathrm{Z}})$ simultaneously. Therefore $\alpha _S(M_{\mathrm{Z}})$ was always fixed to 0.1176 in the original HERAPDF1.0 derivation. When the CMS inclusive jet data are added, this constraint can be dropped and $\alpha _S(M_{\mathrm{Z}})$ and its uncertainty (without *Q* scale variations) is determined to $\alpha _S(M_{\mathrm{Z}}) = 0.1192^{+0.0023}_{-0.0019}\,\text {(all except scale)}$. Repeating the fit with the regularised MC method gives $\alpha _S(M_{\mathrm{Z}}) = 0.1188\pm 0.0041\,\text {(all except scale)}$.

Since a direct correspondence among the different components of the uncertainty can not easily be established, only the quadratic sum of experimental, PDF, and NP uncertainties are presented, which is equivalent to the total uncertainty without scale uncertainty. For example, the HERA-I DIS data contribute to the experimental uncertainty in the combined fits, but contribute only to the PDF uncertainty in separate $\alpha _S(M_{\mathrm{Z}})$ fits. The HERAPDF prescription for PDF fits tends to small uncertainties, while the uncertainties of the MC method with data-derived regularisation are twice as large. For comparison, the corresponding uncertainty in $\alpha _S(M_{\mathrm{Z}})$ using more precisely determined PDFs from global fits as in Sect. 4 gives a result between the two: $\alpha _S(M_{\mathrm{Z}}) = 0.1185\pm 0.0034\,\text {(all except scale)}$.

The evaluation of scale uncertainties is an open issue, which is ignored in all global PDF fits given in Table 1. The impact is investigated in Refs. [20, 76–78], where scale definitions and *K*-factors are varied. Lacking a recommended procedure for the scale uncertainties in combined fits of PDFs and $\alpha _S(M_{\mathrm{Z}})$, two evaluations are reported here for the HERAPDF method. In the first one, the combined fit of PDFs and $\alpha _S(M_{\mathrm{Z}})$ is repeated for each variation of the scale factors from the default choice of $\mu _r =\mu _f =p_{\mathrm {T}}$ for the same six combinations as explained in Sect. 4.2. The scale for the HERA DIS data is not changed. The maximal observed upward and downward changes of $\alpha _S(M_{\mathrm{Z}})$ with respect to the default scale factors are then taken as scale uncertainty, irrespective of changes in the PDFs: $\Delta \alpha _S(M_{\mathrm{Z}}) =\,^{+0.0022}_{-0.0009}\,\mathrm {(scale)}$.

The second procedure is analogous to the method employed to determine $\alpha _S(M_{\mathrm{Z}})$ in Sect. 4. The best PDFs are derived for a series of fixed values of $\alpha _S(M_{\mathrm{Z}})$ as done for the global PDF sets. Using this series of PDFs with varying values of $\alpha _S(M_{\mathrm{Z}})$, the combination of PDF and $\alpha _S(M_{\mathrm{Z}})$ that best fits the HERA-I DIS and CMS inclusive jet data is found. The $\alpha _S(M_{\mathrm{Z}})$ values determined both ways are consistent with each other. The fits are now repeated for the same scale factor variations, and the maximal observed upward and downward changes of $\alpha _S(M_{\mathrm{Z}})$ with respect to the default scale factors are taken as scale uncertainty: $\Delta \alpha _S(M_{\mathrm{Z}}) =\,^{+0.0024}_{-0.0039}\,\mathrm {(scale)}$.

In contrast to the scale uncertainty of the first procedure, there is less freedom for compensating effects between different gluon distributions and $\alpha _S(M_{\mathrm{Z}})$ values in the second procedure, and the latter procedure leads to a larger scale uncertainty as expected. In overall size the uncertainty is similar to the final results on $\alpha _S(M_{\mathrm{Z}})$ reported in the last section: $\Delta \alpha _S(M_{\mathrm{Z}}) =\,^{+0.0053}_{-0.0024}\,\mathrm {(scale)}$.

## Summary

An extensive QCD study has been performed based on the CMS inclusive jet data in Ref. [1]. Fits dedicated to determine $\alpha _S(M_{\mathrm{Z}})$ have been performed involving QCD predictions at NLO complemented with electroweak and NP corrections. Employing global PDFs, where the gluon is constrained through data from various experiments, the strong coupling constant has been determined to be

$$\begin{aligned} \alpha _S(M_{\mathrm{Z}})&= 0.1185 \pm 0.0019\,(\text {exp}) \pm 0.0028\,(\mathrm {PDF})\\&\quad \pm 0.0004\,(\mathrm {NP})^{+0.0053}_{-0.0024}\,(\text {scale}), \end{aligned}$$

which is consistent with previous results.

It was found that the published correlations of the experimental uncertainties adequately reflect the detector characteristics and reliable fits of standard model parameters could be performed within each rapidity region. However, when combining several rapidity regions, it was discovered that the assumption of full correlation in rapidity *y* had to be revised for one source of uncertainty in the JES, which suggested a modified correlation treatment that is described and applied in this work.

To check the running of the strong coupling, all fits have also been carried out separately for six bins in inclusive jet $p_{\mathrm {T}}$, where the scale *Q* of $\alpha _S(Q)$ is identified with $p_{\mathrm {T}}$. The observed behaviour of $\alpha _S(Q)$ is consistent with the energy scale dependence predicted by the renormalization group equation of QCD, and extends the H1, ZEUS, and D0 results to the $\,\text {TeV}$ region.

The impact of the inclusive jet measurement on the PDFs of the proton is investigated in detail using the HERAFitter tool. When the CMS inclusive jet data are used together with the HERA-I DIS measurements, the uncertainty in the gluon distribution is significantly reduced for fractional parton momenta $x \gtrsim 0.01$. Also, a modest improvement in uncertainty in the u and d valence quark distributions is observed.

The inclusion of the CMS inclusive jet data also allows a combined fit of $\alpha _S(M_{\mathrm{Z}})$ and of the PDFs, which is not possible with the HERA-I inclusive DIS data alone. The result is consistent with the reported values of $\alpha _S(M_{\mathrm{Z}})$ obtained from fits employing global PDFs.

## Figures and Tables

**Fig. 18:**
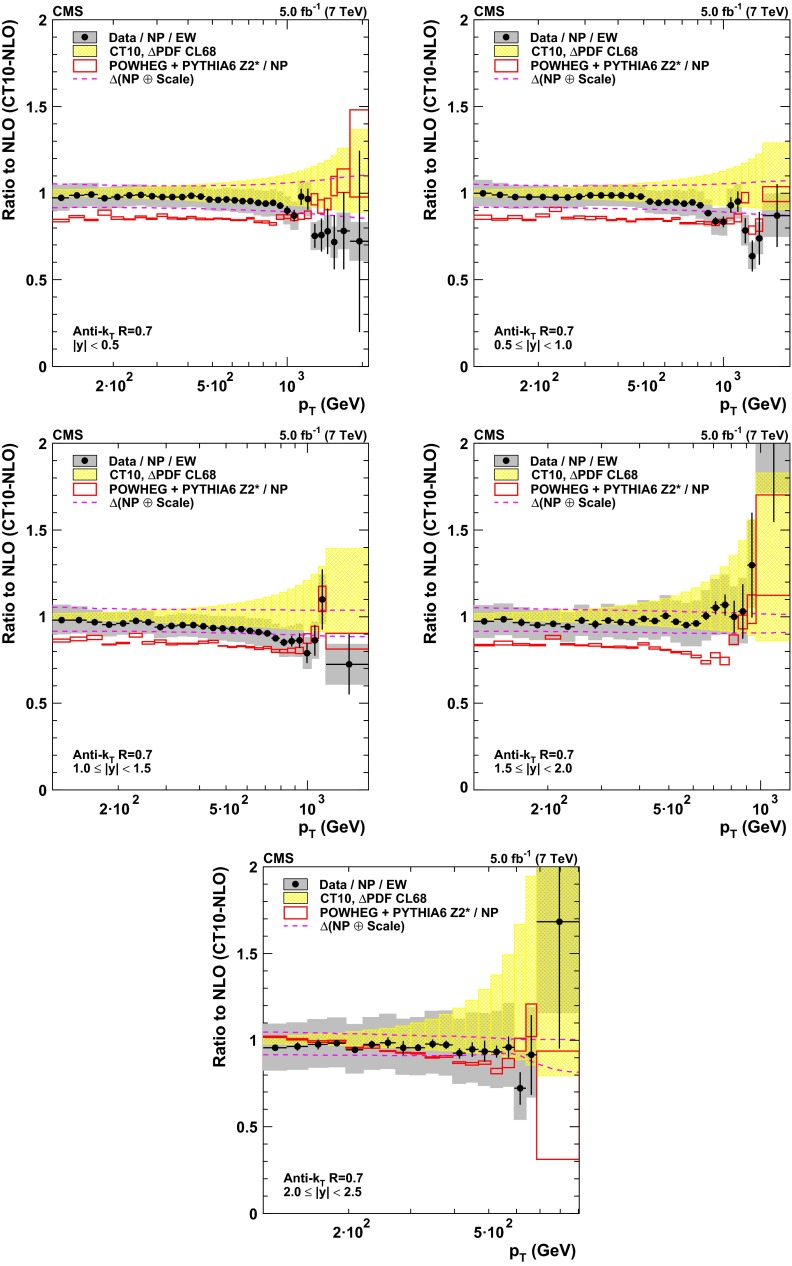
Ratio of data to pQCD at NLO with the CT10-NLO PDF set multiplied by electroweak and NP corrections for the five bins in rapidity together with bands representing the CT10 PDF uncertainty (*hatched*), and the quadratically added scale and NP uncertainty (*dashed lines*). In addition, the ratio of the prediction by powheg $+$ pythia6 tune Z2* at particle level is shown with *boxes* indicating the statistical uncertainty. The *error bars* and the *grey boxes* correspond to the statistical and systematic uncertainty in the data

## Appendix A: Sources of uncertainty in the calibration of jet energies in CMS

In the following, the full list of uncertainty sources of the jet energy calibration procedure that were originally considered by CMS and that were used in Ref. [1] is presented including a short description. It is recommended to apply the procedure with updated correlations for the JEC2 source, as described in Sect. 2.3. A general description of the jet energy calibration procedure of CMS is given in Ref. [10].

When simulations were employed, the following event generators have been used: pythia version 6.4.22 [26] tune Z2 and herwig++ version 2.4.2 [27] with the default tune of version 2.3.

JEC0: Absolute uncertainty. Using data with photon $+$ jet and *Z* $+$ jet events an absolute calibration of jet energies is performed in the jet $p_{\mathrm {T}}$ range of 30–600$\,\text {GeV}$. Uncertainties in the determination of electromagnetic energies in the ECAL, of the muon momenta from $Z\rightarrow \mu \mu$ decays, and of the corrections for initial- and final-state (ISR and FSR) radiation are propagated together with the statistical uncertainty to give the absolute JES uncertainty.
JEC1: High- and low-$p_{\mathrm {T}}$ extrapolation uncertainty. Where an absolute calibration with data is not possible, events are produced with the event generators pythia6 and herwig++ and are subsequently processed through the CMS detector simulation based on Geant4 [79]. Differences in particular in modelling the fragmentation process and the underlying event lead to an extrapolation uncertainty relative to the directly calibrated jet $p_{\mathrm {T}}$ range of 30–600$\,\text {GeV}$.
JEC2: High-$p_{\mathrm {T}}$ extrapolation uncertainty. This source accounts for a $\pm 3$ % variation in the single-particle response that is propagated to jets using a parameterized fast simulation of the CMS detector [80].
JEC3: Jet flavour related uncertainty. Differences in detector response to light, charm, and bottom quark as well as gluon jets relative to the mixture predicted by QCD for the measured processes are evaluated on the basis of simulations with pythia6 and herwig++.
JEC4: Uncertainty caused by time dependent detector effects. This source considers residual time-dependent variations in the detector conditions such as the endcap ECAL crystal transparency.
JEC5–JEC10: $\eta$-dependent uncertainties coming from the dijet balance method:
JEC5–JEC7: Caused by the jet energy resolution. These three sources are assumed to be fully correlated for the endcap with upstream tracking detectors (JEC5), the endcap without upstream tracking detectors (JEC6), and the HF calorimeter (JEC7).
JEC8: $\eta$-dependent uncertainty caused by corrections for final-state radiation. The uncertainty is correlated from one region to the other and increases towards HF.
JEC9–JEC10: Statistical uncertainty in the determination of $\eta$-dependent corrections. These are two separate sources for the endcap without upstream tracking detectors (JEC9), and the HF calorimeter (JEC10).
JEC11–JEC15: Uncertainties for the pileup corrections:
JEC11: Parameterizes differences between data and MC events versus $\eta$ in zero-bias data.
JEC12: Estimates residual out-of-time pileup for prescaled triggers, if MC events are reweighted to unprescaled data.
JEC13: Covers an offset dependence on jet $p_{\mathrm {T}}$ (due to, e.g. zero-suppression effects), when the correction is calibrated for jets in the $p_{\mathrm {T}}$ range of 20–30$\,\text {GeV}$.
JEC14: Accounts for differences in measured offset from zero-bias MC events and from generator-level information in a QCD sample.
JEC15: Covers observed jet rate variations versus the average number of reconstructed primary vertices in the 2011 single-jet triggers after applying L1 corrections.

## Appendix B: Comparison to theoretical predictions by powheg$+$pythia6

Figure 18 presents ratios of data over theory predictions at NLO using the CT10-NLO PDF set multiplied by electroweak and NP corrections including PDF uncertainties.

## References

[1] CMS Collaboration, Measurements of differential jet cross sections in proton–proton collisions at $$\sqrt{s}=7$$ TeV with the CMS detector. Phys. Rev. D **87**, 112002 (2013). doi:10.1103/PhysRevD.87.112002. arXiv:1212.6660

[2] ATLAS Collaboration, Measurement of the inclusive jet cross section in $$pp$$ collisions at $$\sqrt{s}=2.76$$ TeV and comparison to the inclusive jet cross section at $$\sqrt{s}=7$$ TeV using the ATLAS detector. Eur. Phys. J. C **73**, 2509 (2013). doi:10.1140/epjc/s10052-013-2509-4. arXiv:1304.4739 10.1140/epjc/s10052-013-2509-4 PMC4400855 25904819

[3] CMS Collaboration, The CMS experiment at the CERN LHC. JINST **3**, S08004 (2008). doi:10.1088/1748-0221/3/08/S08004

[4] Cacciari M, Salam GP, Soyez G (2008). The anti-$$k_t$$ jet clustering algorithm. JHEP.

[5] Cacciari M, Salam GP, Soyez G (2012). FastJet user manual. Eur. Phys. J. C.

[6] CMS Collaboration, Jet Energy Scale performance in 2011. CMS Detector Performance Summary CMS-DP-2012/006

[7] CMS Collaboration, Absolute calibration of the luminosity measurement at CMS: winter 2012 update. CMS Physics Analysis Summary CMS-PAS-SMP-12-008

[8] D’Agostini G (1995). A multidimensional unfolding method based on Bayes’ theorem. Nucl. Instrum. Methods A.

[9] T. Adye, Unfolding algorithms and tests using RooUnfold, in *Proceedings of the PHYSTAT 2011 Workshop on Statistical Issues Related to Discovery Claims in Search Experiments and Unfolding* (CERN, Geneva, 2011), p. 313. doi:10.5170/CERN-2011-006. arXiv:1105.1160

[10] CMS Collaboration, Determination of jet energy calibration and transverse momentum resolution in CMS. JINST **6**, P11002 (2011). doi:10.1088/1748-0221/6/11/P11002. arXiv:1107.4277

[11] J. Pumplin et al., Uncertainties of predictions from parton distribution functions. II: the Hessian method. Phys. Rev. D **65**, 014013 (2001). doi:10.1103/PhysRevD.65.014013. arXiv:hep-ph/0101032

[12] J. Butterworth et al., Les Houches 2013: physics at TeV colliders: standard model working group report (2014). arXiv:1405.1067

[13] Dittmaier S, Huss A, Speckner C (2012). Weak radiative corrections to dijet production at hadron colliders. JHEP.

[14] Z. Nagy, Three-jet cross sections in hadron–hadron collisions at next-to-leading order. Phys. Rev. Lett. **88**, 122003 (2002). doi:10.1103/PhysRevLett.%2088.122003. arXiv:hep-ph/0110315 10.1103/PhysRevLett.88.122003 11909448

[15] Z. Nagy, Next-to-leading order calculation of three-jet observables in hadron–hadron collisions. Phys. Rev. D **68**, 094002 (2003). doi:10.1103/PhysRevD.68.094002. arXiv:hep-ph/0307268

[16] D. Britzger, K. Rabbertz, F. Stober, M. Wobisch, New features in version 2 of the fastNLO project, in *20th International Workshop on Deep-Inelastic Scattering and Related Subjects (DIS’12)*, Bonn, p. 217 (2012). doi:10.3204/DESY-PROC-2012-02/165. arXiv:1208.3641

[17] Alekhin S, Blümlein J, Moch S (2012). Parton distribution functions and benchmark cross sections at next-to-next-to-leading order. Phys. Rev. D.

[18] Lai H-L (2010). New parton distributions for collider physics. Phys. Rev. D.

[19] H1 and ZEUS Collaboration, Combined measurement and QCD analysis of the inclusive $$e^\pm p$$ scattering cross sections at HERA. JHEP **01**, 109 (2010). doi:10.1007/JHEP01(2010)%20109. arXiv:0911.0884

[20] Martin AD, Stirling WJ, Thorne RS, Watt G (2009). Parton distributions for the LHC. Eur. Phys. J. C.

[21] Martin AD, Stirling WJ, Thorne RS, Watt G (2009). Uncertainties on $$\alpha _S$$ in global PDF analyses and implications for predicted hadronic cross sections. Eur. Phys. J. C.

[22] NNPDF Collaboration, Impact of heavy quark masses on parton distributions and LHC Phenomenology. Nucl. Phys. B **849**, 296 (2011). doi:10.1016/j.nuclphysb.2011.03.021. arXiv:1101.1300

[23] Marchesini G, Webber BR (1988). Monte Carlo simulation of general hard processes with coherent QCD radiation. Nucl. Phys. B.

[24] Knowles IG (1990). A linear algorithm for calculating spin correlations in hadronic collisions. Comput. Phys. Commun..

[25] Knowles IG (1988). Spin correlations in Parton–Parton scattering. Nucl. Phys. B.

[26] T. Sjöstrand, S. Mrenna, P. Skands, PYTHIA 6.4 physics and manual. JHEP **05**, 026 (2006). doi:10.1088/1126-6708/2006/05/026. arXiv:hep-ph/0603175

[27] Bähr M (2008). Herwig++ physics and manual. Eur. Phys. J. C.

[28] Sjöstrand T, van Zijl M (1987). A multiple interaction model for the event structure in hadron collisions. Phys. Rev. D.

[29] Bähr M, Gieseke S, Seymour MH (2008). Simulation of multiple partonic interactions in Herwig++. JHEP.

[30] Andersson B, Gustafson G, Ingelman G, Sjöstrand T (1983). Parton fragmentation and string dynamics. Phys. Rep..

[31] Andersson B, Gustafson G, Soderberg B (1983). A general model for jet fragmentation. Z. Phys. C.

[32] Sjöstrand T (1984). The merging of jets. Phys. Lett. B.

[33] Webber BR (1984). A QCD model for jet fragmentation including soft gluon interference. Nucl. Phys. B.

[34] Frixione S, Nason P, Oleari C (2007). Matching NLO QCD computations with Parton Shower simulations: the POWHEG method. JHEP.

[35] Alioli S, Nason P, Oleari C, Re E (2010). A general framework for implementing NLO calculations in shower Monte Carlo programs: the POWHEG BOX. JHEP.

[36] Alioli S (2011). Jet pair production in POWHEG. JHEP.

[37] J.M. Campbell, J.W. Huston, W.J. Stirling, Hard Interactions of Quarks and Gluons: A Primer for LHC Physics. Rept. Prog. Phys. **70**, 89 (2007). doi:10.1088/0034-4885/70/1/R02. arXiv:hep-ph/0611148

[38] R. Field, Early LHC underlying event data – findings and surprises, in *21st Hadron Collider Physics Symposium (HCP’10)*, Toronto (2010). arXiv:1010.3558

[39] J. Pumplin et al., New generation of parton distributions with uncertainties from global QCD analysis. JHEP **07**, 012 (2002). doi:10.1088/1126-6708/2002/07/012. arXiv:hep-ph/0201195

[40] H.L. Lai et al., Global QCD analysis of parton structure of the nucleon: CTEQ5 parton distributions. Eur. Phys. J. C **12**, 375 (2000). doi:10.1007/s100529900196. arXiv:hep-ph/9903282

[41] Buckley A (2011). General-purpose event generators for LHC physics. Phys. Rep..

[42] P. Nason, A new method for combining NLO QCD with shower Monte Carlo algorithms. JHEP **11**, 040 (2004). doi:10.1088/1126-6708/2004/11/040. arXiv:hep-ph/0409146

[43] Dooling S, Gunnellini P, Hautmann F, Jung H (2013). Longitudinal momentum shifts, showering, and nonperturbative corrections in matched next-to-leading-order shower event generators. Phys. Rev. D.

[44] Skands PZ (2010). Tuning Monte Carlo generators: the Perugia tunes. Phys. Rev. D.

[45] Lyons L, Martin AJ, Saxon DH (1990). On the determination of the $$B$$ lifetime by combining the results of different experiments. Phys. Rev. D.

[46] D’Agostini G (2003). Bayesian Reasoning in Data Analysis: A Critical Introduction.

[47] NNPDF Collaboration, Fitting parton distribution data with multiplicative normalization uncertainties, JHEP **05**, 075 (2010). doi:10.1007/JHEP05(2010)075. arXiv:0912.2276

[48] NNPDF Collaboration, A first unbiased global NLO determination of parton distributions and their uncertainties. Nucl. Phys. B **838**, 136 (2010). doi:10.1016/j.nuclphysb.2010.05.008. arXiv:1002.4407

[49] Salam GP, Rojo J (2009). A higher order perturbative parton evolution toolkit (HOPPET). Comput. Phys. Commun..

[50] Particle Data Group, K.A. Olive et al., Review of particle physics. Chin. Phys. C **38**, 090001 (2014). doi:10.1088/1674-1137/38/9/090001

[51] CDF Collaboration, Measurement of the strong coupling constant from inclusive jet production at the Tevatron $$\bar{p}p$$ collider. Phys. Rev. Lett. **88**, 042001 (2002). doi:10.1103/PhysRevLett.88.042001. arXiv:hep-ex/0108034 10.1103/PhysRevLett.88.042001 11801109

[52] D0 Collaboration (2009). Determination of the strong coupling constant from the inclusive jet cross section in $$p\bar{p}$$ collisions at $$\sqrt{s}=1.96$$ TeV. Phys. Rev. D.

[53] D0 Collaboration (2012). Measurement of angular correlations of jets at $$\sqrt{s}=1.96$$ TeV and determination of the strong coupling at high momentum transfers. Phys. Lett. B.

[54] Malaescu B, Starovoitov P (2012). Evaluation of the strong coupling constant $$\alpha _S$$ using the ATLAS inclusive jet cross-section data. Eur. Phys. J. C.

[55] CMS Collaboration, Measurement of the ratio of the inclusive 3-jet cross section to the inclusive 2-jet cross section in pp collisions at $$\sqrt{s}$$ = 7 TeV and first determination of the strong coupling constant in the TeV range. Eur. Phys. J. C **73**, 2604 (2013). doi:10.1140/epjc/s10052-013-2604-6. arXiv:1304.7498

[56] CMS Collaboration, Determination of the top-quark pole mass and strong coupling constant from the $${\rm t}{\bar{\rm t}}$$ production cross section in pp collisions at TEV $$\sqrt{s} = 7$$. Phys. Lett. B **728**, 496 (2014). doi:10.1016/j.physletb.2013.12.009

[57] H1 Collaboration, Jet production in ep collisions at High $$Q^2$$ and determination of $$\alpha _s$$. Eur. Phys. J. C **65**, 363 (2010). doi:10.1140/epjc/s10052-009-1208-7. arXiv:0904.3870

[58] H1 Collaboration, Jet production in ep collisions at Low $$Q^2$$ and determination of $$\alpha _s$$. Eur. Phys. J. C **67**, 1 (2010). doi:10.1140/epjc/s10052-010-1282-x. arXiv:0911.5678

[59] H1 Collaboration, Measurement of multijet production in ep collisions at high $$Q^2$$ and determination of the strong coupling $$\alpha _s$$ (2014). arXiv:1406.4709. (Submitted to Eur. Phys. J. C)

[60] ZEUS Collaboration, Inclusive-jet photoproduction at HERA and determination of $$\alpha _s$$. Nucl. Phys. B **864**, 1 (2012). doi:10.1016/j.nuclphysb.2012.06.006. arXiv:1205.6153

[61] S. Alekhin et al., HERAFitter, open source QCD fit project (2014). arXiv:1410.4412. (Submitted to Eur. Phys. J. C)

[62] HERAFitter web site. http://www.herafitter.org. Accessed 20 June 2015

[63] NNPDF Collaboration, A determination of parton distributions with faithful uncertainty estimation. Nucl. Phys. B **809**, 1 (2009). doi:10.1016/j.nuclphysb.2008.09.037. arXiv:0808.1231

[64] Gribov VN, Lipatov LN (1972). Deep inelastic ep scattering in perturbation theory. Sov. J. Nucl. Phys..

[65] Altarelli G, Parisi G (1977). Asymptotic freedom in parton language. Nucl. Phys. B.

[66] Dokshitzer YL (1977). Calculation of the structure functions for deep inelastic scattering and e$$+$$ e$$-$$ annihilation by perturbation theory in quantum chromodynamics. Sov. Phys. JETP.

[67] Botje M (2011). QCDNUM: fast QCD evolution and convolution. Comput. Phys. Commun..

[68] R.S. Thorne, R.G. Roberts, An ordered analysis of heavy flavour production in deep inelastic scattering. Phys. Rev. D **57**, 6871 (1998). doi:10.1103/PhysRevD.57.6871. arXiv:hep-ph/9709442

[69] R.S. Thorne, Variable-flavor number scheme for next-to-next-to-leading order. Phys. Rev. D **73**, 054019 (2006). doi:10.1103/PhysRevD.73.054019. arXiv:hep-ph/0601245

[70] ATLAS Collaboration, Determination of the strange quark density of the proton from ATLAS measurements of the $$W \rightarrow \ell \nu $$ and $$Z \rightarrow \ell \ell $$ cross sections. Phys. Rev. Lett. **109**, 012001 (2012). doi:10.1103/PhysRevLett.109.012001. arXiv:1203.4051 10.1103/PhysRevLett.109.012001 23031098

[71] ATLAS Collaboration, Measurement of the low-mass Drell–Yan differential cross section at $$\sqrt{s} = 7$$ TeV using the ATLAS detector. JHEP **06**, 112 (2014). doi:10.1007/JHEP06(2014)112. arXiv:1404.1212

[72] H1 and ZEUS Collaboration, Combination and QCD analysis of charm production cross section measurements in deep-inelastic ep scattering at HERA. Eur. Phys. J. C **73**, 2311 (2013). doi:10.1140/epjc/s10052-013-2311-3. arXiv:1211.1182

[73] Collaboration NuTeV (2007). Measurement of the Nucleon Strange-Antistrange Asymmetry at Next-to-Leading Order in QCD from nucleon strange-antistrange asymmetry at next-to-leading order in QCD from NuTeV Dimuon data. Phys. Rev. Lett..

[74] D. Stump et al., Uncertainties of predictions from parton distribution functions I: The Lagrange multiplier method. Phys. Rev. D **65**, 014012 (2001). doi:10.1103/PhysRevD.65.014012. arXiv:hep-ph/0101051

[75] M. Botje, Error estimates on parton density distributions. J. Phys. G **28**, 779 (2002). doi:10.1088/0954-3899/28/5/305. arXiv:hep-ph/0110123

[76] Gao J (2013). MEKS: a program for computation of inclusive jet cross sections at hadron colliders. Comput. Phys. Commun..

[77] Ball RD (2013). Parton distribution benchmarking with LHC data. JHEP.

[78] Watt BJA, Motylinski P, Thorne RS (2014). The effect of LHC jet data on MSTW PDFs. Eur. Phys. J. C.

[79] GEANT4 Collaboration, GEANT4 – a simulation toolkit. Nucl. Instrum. Methods A **506**, 250 (2003). doi:10.1016/S0168-9002(03)01368-8

[80] CMS Collaboration, The fast simulation of the CMS detector at LHC. J. Phys. Conf. Ser. **331**, 032049 (2011). doi:10.1088/1742-6596/331/3/032049

